# Liquid-liquid phase separation in gastric cancer: identifying novel biomarkers and therapeutic targets through gene signature analysis

**DOI:** 10.3389/fimmu.2025.1620390

**Published:** 2025-09-01

**Authors:** Xianhui Wen, Miaomiao Cui, Junhua Zhang, Hai Huang

**Affiliations:** ^1^ Center for Clinical Laboratories, The Affiliated Hospital of Guizhou Medical University, Guiyang, China; ^2^ School of Clinical Laboratory Science, Guizhou Medical University, Guiyang, China; ^3^ Department of Clinical Laboratory, The Second People’s Hospital of Guiyang, Guiyang, China; ^4^ Department of Blood Transfusion, The Third Xiangya Hospital Central South University, Changsha, China

**Keywords:** gastric cancer, liquid-liquid phase separation, tumor immune microenvironment, prognosis, nomogram

## Abstract

**Background and objective:**

Liquid-liquid phase separation (LLPS) plays an important role in the development of many tumors, including gastric cancer, but its prognostic value is unclear. The aim of this study was to explore the prognostic significance of LLPS-related genes in gastric cancer to provide a basis for improving the accuracy of prognostic prediction and finding potential therapeutic targets in gastric cancer.

**Methods:**

Clinical and transcriptomic data of gastric cancer were downloaded from TCGA and GEO databases, and LLPS-related genes were extracted from PhaSepDB. Unsupervised clustering was used to identify molecular subtypes based on LLPS gene expression. LLPS gene features were constructed and validated by LASSO Cox regression, and their staging prediction value was also evaluated by machine learning methods. Key genes were validated by qRT-PCR, Western blot, immunofluorescence, and functional experiments (shRNA knockdown, CCK-8, clone formation, and scratch assay).

**Results:**

Twenty LLPS-associated genes showed significant mRNA expression, copy number variation, somatic mutation, and interaction network alterations in gastric cancer tissues. Two LLPS molecular isoforms with different survival outcomes and immune microenvironment characteristics were identified. A four-gene LLPS prognostic signature consisting of *DACT1, EZH2, PAK2*, and *PSPC1* was constructed, and the high-risk group had a poorer prognosis and was prone to drug resistance. Machine learning analysis further confirmed the predictive value of this gene signature. Functional experiments showed that knockdown of PSPC1 significantly inhibited the proliferation (inhibition rate >50%, *P <*0.001) and migration ability (*P*<0.0001) of gastric cancer cells. Immunofluorescence confirmed the localization and aggregation characteristics of DACT1 and PSPC1.

**Conclusion:**

This study revealed the important role of LLPS in gastric cancer, and the constructed four-gene LLPS signature is expected to be a novel biomarker for prognostic assessment and treatment of gastric cancer. PSPC1 plays a key role in gastric cancer progression, and has the value of a potential therapeutic target.

## 1 Introduction

GC ranks as the fifth most common malignancy and is the fourth leading cause of cancer-related death worldwide (1). Despite its high occurrence, a large proportion of patients are unfortunately diagnosed at more advanced stages, resulting in poor clinical outcomes due to the lack of clear clinical markers (2). Among patients with locoregionally confined GC, the 5-year relative overall survival rate is 77.7%, but for those with advanced cancer, it drops to just 10.2% (3). Therefore, discovering new prognostic biomarkers and potential therapeutic targets is essential for better patient outcomes.

LLPS is a physicochemical process within cells that has gained significant attention in recent years (4). LLPS results in the formation of membraneless, droplet-like structures in the cytoplasm or nucleoplasm, creating dynamic microenvironments that regulate various biological processes (5, 6). LLPS is driven by multivalent interactions among macromolecules, with one key mechanism involving the intrinsically disordered regions (IDRs) of proteins (7). Increasing evidence indicates that LLPS plays a crucial role in cancer initiation (8, 9), progression (10), immune escape (11, 12), vascularization (13, 14), metabolism, phenotypic plasticity (15), and metastasis (16, 17). However, the role of LLPS in GC remains insufficiently understood and requires further in-depth investigation.

Based on this background, this study aimed to systematically identify and assess the importance of LLPS-associated genes in GC through comprehensive bioinformatics analysis. We developed an innovative molecular signature centered on LLPS-related genes, offering clinically actionable tools for personalized treatment and prognosis assessment of GC, and laying the groundwork for a deeper understanding of LLPS’s role in GC development and progression.

## 2 Materials and methods

### 2.1 Ethical approval

All data usage complied strictly with the relevant data use policies and ethical governance frameworks of the TCGA and GEO databases. All analyses were conducted using anonymized public data.

### 2.2 Data acquisition and preliminary processing

**Figure 1:**
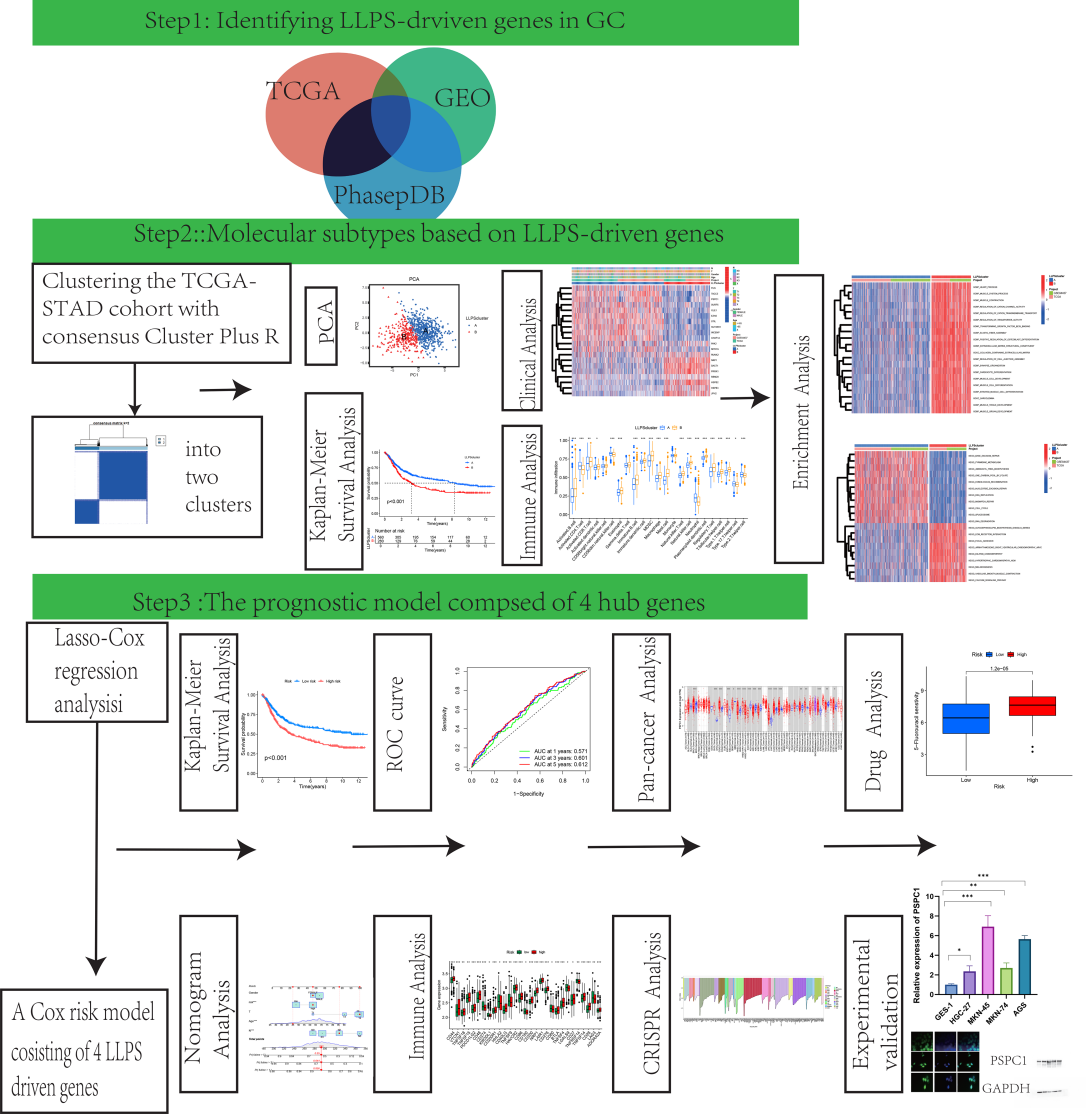
The flow diagram of our study.

**Figure 1** provided a schematic overview of the study’s design and methodological flowchart. Transcriptomic data and clinically annotated patient information were sourced from the TCGA-STAD project and the GSE84437 dataset available in the GEO database. In August 2024, we obtained the TCGA-STAD data from the official TCGA portal (https://portal.gdc.cancer.gov/), which comprises RNA sequencing and clinical information for 412 gastric adenocarcinoma patients and 36 normal tissues on August 15, 2024. The GSE84437 dataset, consisting of data from 433 GC patients, was retrieved from the GEO database (https://www.ncbi.nlm.nih.gov/geo/query/acc.cgi?acc=GSE84437). Survival records for 448 patients were extracted from the TCGA dataset, and the analysis included only those patients with complete gene expression and survival data. The 561 LLPS-related genes were sourced from PhaSepDB (http://db.phasep.pro/) (18). GSE19826 and GSE79973 datasets as validation sets.

Before merging the TCGA and GEO datasets, batch effect correction was performed using the ComBat function from the “sva” R package to minimize technical variation between different data sources. The corrected data were then used for subsequent differential expression analysis and model construction.

### 2.3 Determination of molecular subtypes

We utilized the “ConsensusClusterPlus” R package to perform unsupervised consensus clustering for patient classification. Subsequently, patient clusters were discerned and verified through Principal Component Analysis (PCA).

### 2.4 Investigating pathological profiles and prognostic patterns across LLPS clusters

We utilized the “survival” and “survminer” packages in R to conduct Kaplan-Meier (K-M) survival analyses, examining the prognostic relevance of GC patients grouped by distinct LLPS-based clusters. Moreover, we assessed clinical variables such as age, tumor stage (T stage), and lymph node stage (N stage) across these clusters to identify any statistically significant associations.

### 2.5 Molecular signature characterization through gene set variation analysis of LLPS clusters

To investigate the underpinning mechanisms of the distinctive LLPS-derived clusters identified in this research, we applied the R package “GSVA”. This method enabled us to assess pathway activity differences associated with the unique LLPS patterns, providing insights into the functional implications of LLPS in GC.

### 2.6 Estimation of the tumor microenvironment in different LLPS clusters

By applying single-sample Gene Set Enrichment Analysis (ssGSEA), we quantified the relative representation of 23 human immune cell populations within the tumor microenvironment (TME) across multiple LLPS clusters. Furthermore, we assessed the transcript abundance of 33 key immune−checkpoint regulators across these clusters to investigate differences in immune profile.

### 2.7 Establishment of a prognostic index derived from a differentially expressed gene model

The study utilized a dataset of 871 GC samples, consisting of 433 samples from the GEO database (GSE84437), noted for its large size and detailed clinical follow-up, and 438 gastric adenocarcinoma samples with survival information from the TCGA database. Utilizing the R−based toolkit “caret” (19), we splited the combined dataset evenly into a training and testing subsets, each comprising 436 patients. The training set underwent univariate Cox regression analysis to find LLPS-related differentially expressed genes (DEGs) correlated with overall survival (OS) (20). We then employed the Least Absolute Shrinkage and Selection Operator (LASSO) algorithm with the R package “glmnet” to choose the DEGs with the highest prognostic potential. Subsequently, multivariate Cox regression analysis was employed to identify independent prognostic DEGs associated with GC, which were then utilized to construct the prognostic model. In conclusion, aligning with findings from previous oncological research (21), the risk−prediction score was determined based on the following equation involving the selected genes:

Prognostic Score = (Gene A expression × Coefficient A) + (Gene B expression × Coefficient B) + …

### 2.8 Independent prognostic analysis of the risk model

To evaluate the independent prognostic significance of the risk signature, univariate and multivariate Cox proportional-hazards regression analyses were carried out utilizing the R package “survival”. These analyses assessed the impact of the risk score and other clinicopathological variables on overall survival. The K-M method was employed to analyze survival outcomes, and survival curves between different prognostic groups were compared using the log-rank test to assess statistical significance.

Furthermore, we constructed a nomogram integrating both clinicopathological characteristics and the prognostic risk score using the”rms”R package. The concordance index (c-index) was computed to evaluate the model’s predictive performance and the agreement between projected survival probabilities and observed outcomes. Furthermore, calibration plots and receiver operating characteristic (ROC) curves were constructed to examine the model’s reliability and predictive accuracy.

### 2.9 TIDE analysis

We applied the TIDE (Tumor Immune Dysfunction and Exclusion) tool to assess tumor immune evasion mechanisms and analyzed the discrepancies in TIDE scores between different risk groups.

### 2.10 Mutation data analysis

We used the R package “maftools” to preprocess and visualize mutation data from TCGA stomach adenocarcinoma samples, including mutation frequency analysis, distribution of mutation types, and waterfall plots of mutated genes.

### 2.11 Investigation of the immunological microenvironment characteristics and pharmacological response profiles

We utilized the ESTIMATE computational framework to quantify stromal and immune cell infiltration levels in gastric carcinoma specimens. Through the R package “estimate”, we systematically generated three quantitative metrics: stromal scores reflecting extracellular matrix components, immune scores representing leukocyte infiltration, and composite ESTIMATE scores. To examine therapeutic response patterns, pharmacological sensitivity data were acquired from the publicly accessible Genomics of Drug Sensitivity in Cancer repository(https://www.cancerrxgene.org/) (22). Spearman rank correla- tion was applied to explore the association between drug−response patterns and the prognostic index. Furthermore, the R computational toolkit “pRRophetic” was implemented to predict half-maximal inhibitory concentrations (IC50), enabling comparative analysis of chemotherapeutic efficacy between prognostic subgroups.

### 2.12 The whole-gene CRISPR-Cas9 screens via the computational estimation of CRISPR effects by relative screen signal

Genome-wide screening CRISPR were downloaded from DepMap database (https://depmap.org/portal/download/). Approximately 17000 candidate genes were calculated by using CERES algorithm the dependence of the score (23). A negative score indicates cell growth inhibition or death following gene knockout, with scores of 0 and -1 representing the median effects of non-essential genes and common core essential genes, respectively. The top 200 negatively scoring genes were visualized in a bar char.

### 2.13 Pan-cancer analysis of gene expression

Gene expression data was derived from the normalized TCGA dataset, with RNA-seq data obtained from the EBPlusPlusAdjust PANCAN_IlluminaHiSeq_RNASeqV2.geneExp.tsv file provided by PanCanAtlas. The data was transformed into dimensionless Z-Score values by tumor using (x-μ)/σ. Z-score values less than -3 or greater than 3 were considered outliers and were removed. After outlier removal, tumors were included in the analysis when there were at least three normal samples. Wilcoxon Rank Sum Tests were used to compare the statistical differences in expression levels between tumor and normal tissues in the digestive system tumor dataset.

### 2.14 Cell culture

The gastric epithelial cell line GES and gastric cancer cell lines HGC-27, MKN-45, MKN-74, and AGS were acquired from the Chinese Academy of Sciences Cell Bank (Shanghai, China). Cells were cultured in RPMI-1640 medium (Gibco, NY, USA) enriched with 10% fetal bovine serum (FBS) (Biological Industries, KBH, IL) and 1% penicillin-streptomycin solution (Gibco, NY, USA). All cell cultures were maintained in incubation vessels at 37 °C in a humidified atmosphere containing 5% CO₂.

### 2.15 Quantitative reverse-transcription polymerase chain reaction validate RNA expression of key genes

GC cells were collected for RNA extraction using TRIzol reagent (Invitrogen, CA, USA). Total RNA was reverse transcribed to cDNA using PrimeScriptTM RT reagent Kit (TaKaRa, Shiga, Japan). Quantitative real-time PCR was performed using ChamQ SYBR qPCR Master Mix (Vazyme, Nanjing, China) according to the manufacturer’s instructions. The relative expression levels were normalized to HPRT and calculated using the 2^–△△Ct^ method. All primer sequences used for RT-qPCR analysis are listed in **Supplementary Table S1**.

### 2.16 Immunofluorescence

Following culture, cells underwent fixation with 4% paraformaldehyde solution (10 min) and membrane permeabilization using 1% Triton X-100 (5 min) at ambient temperature. To prevent non-specific interactions, cells were immersed in a 5% BSA solution for 1 h at ambient temperature. The samples were then exposed to specific primary antibodies and maintained at 4°C for 12 hours to ensure complete reaction equilibrium. After thorough PBS washing steps, samples were treated with goat anti-mouse secondary antibodies conjugated to Alexa Fluor 488 (Thermo Fisher Scientific) for one hour under ambient conditions. Nuclear visualization was achieved through DAPI counterstaining. Immunofluorescence(IF) images were acquired using a DMi8 LEICA fluorescence microscope system. **Supplementary Table S2** presents the full set of primary antibodies utilized in this study.

### 2.17 Western blot analysis

Protein samples were isolated through RIPA buffer-mediated lysis (Solarbio, Beijing, China). Following protein separation through SDS-PAGE electrophoresis, the samples were transferred to PVDF membranes (Millipore, MA, USA). Subsequently, the membranes underwent blocking with 5% non-fat dry milk solution in TBST buffer at ambient temperature for 2 h. After blocking, membranes were incubated overnight at 4 °C with the designated primary antibodies, then exposed for 1h at room temperature to the matching HRP−conjugated secondary antibodies. Immunoreactive bands were detected using an enhanced chemiluminescence substrate (MeilunBio, Dalian, China) and documented using a ChemiDoc XRS+ system (Bio-Rad, CA, USA). Detailed information regarding the primary antibodies utilized is available in **Supplementary Table S2**.

### 2.18 Lentivirus production and generation of stable cell lines

Short hairpin RNAs (shRNAs) targeting human PSPC1 were purchased from Zhenjiang Huamao Biotechnology Co., Ltd. (Zhenjiang, China) and supplied in the lentiviral vector pLenti-U6-shRNA-CMV- GFP-2A-Puro. Silencing were generated in HEK293T cells co-transfected with the PSPAX2 plasmid and PMD2G plasmid via Polyethylenimine(PEI) transfection reagent (Solarbio,China). Viral supernatants were collected at 48h and 72h post-transfection, filtered using a 0.45μm pore-size membrane, and enriched with 10% PEG-6000. Target cells were transduced with the lentiviral preparations in the presence of 8 mg/mL polybrene. At 48h later, puromycin (Solarbio,China) was added to a final concentration of 2 μg/mL for selection, alongside a negative-selection control group at the same density. Selection was discontinued once all cells in the control dish had died, and the surviving population was expanded as a stably transduced line.

### 2.19 CCK8 assay

HGC-27 and AGS cells were seeded at a density of 3×10³ cells per well. Each well received 10μL of CCK-8 solution (MCE, Shanghai, China). Following an additional 2-hour incubation period, the optical density at 450 nm was determined using a microplate reader (Thermo Fisher Scientific, MA, USA).

### 2.20 Colony formation assay

For colony formation analysis, HGC-27 and AGS cells were plated in 6-well plates at 1×10^3^ cells per well. After 10 days, colonies were fixed with 4% paraformaldehyde and stained using 0.1% crystal violet solution. The number of colonies formed was counted using Image J software.

### 2.21 Wound healing assay

AGS cells were transferred to 6-well plates. Following 24 hours of incubation to allow cell attachment, a scratch wound was generated in the cell monolayer using a sterile 200μL pipette tip. The culture medium was then replaced with medium containing 1% FBS instead of 10% FBS. Cell migration was monitored by capturing images at various time points, and the wound area was quantified using Image J software.

### 2.22 Statistical analysis

Comprehensive data assessments were performed through the R environment (version 4.4.0) and validated using GraphPad Prism (version 9.0) for all statistical computations. For continuous variables, the Student’s t-test was applied; for categorical variables, the Chi-square test or Fisher’s exact test was used. By applying the “limma” R package for differential expression analysis, significant differences were identified using criteria of FDR<0.05 combined with |log₂−fold change|>1. Survival analysis was conducted using the “survival” and “survminer” R packages, with the K-M method employed to calculate survival functions and the log-rank test used to compare survival curves between different prognostic groups. In cases involving multiple comparisons, the Benjamini-Hochberg correction was employed to maintain the false discovery rate (FDR) at an acceptable level. All p-values were founded on two-sided tests, and results with p-values below 0.05 were considered statistically significant.

## 3 Results

### 3.1 Panoramic profiling of LLPS-linked genetic features in GC

**Figure 2:**
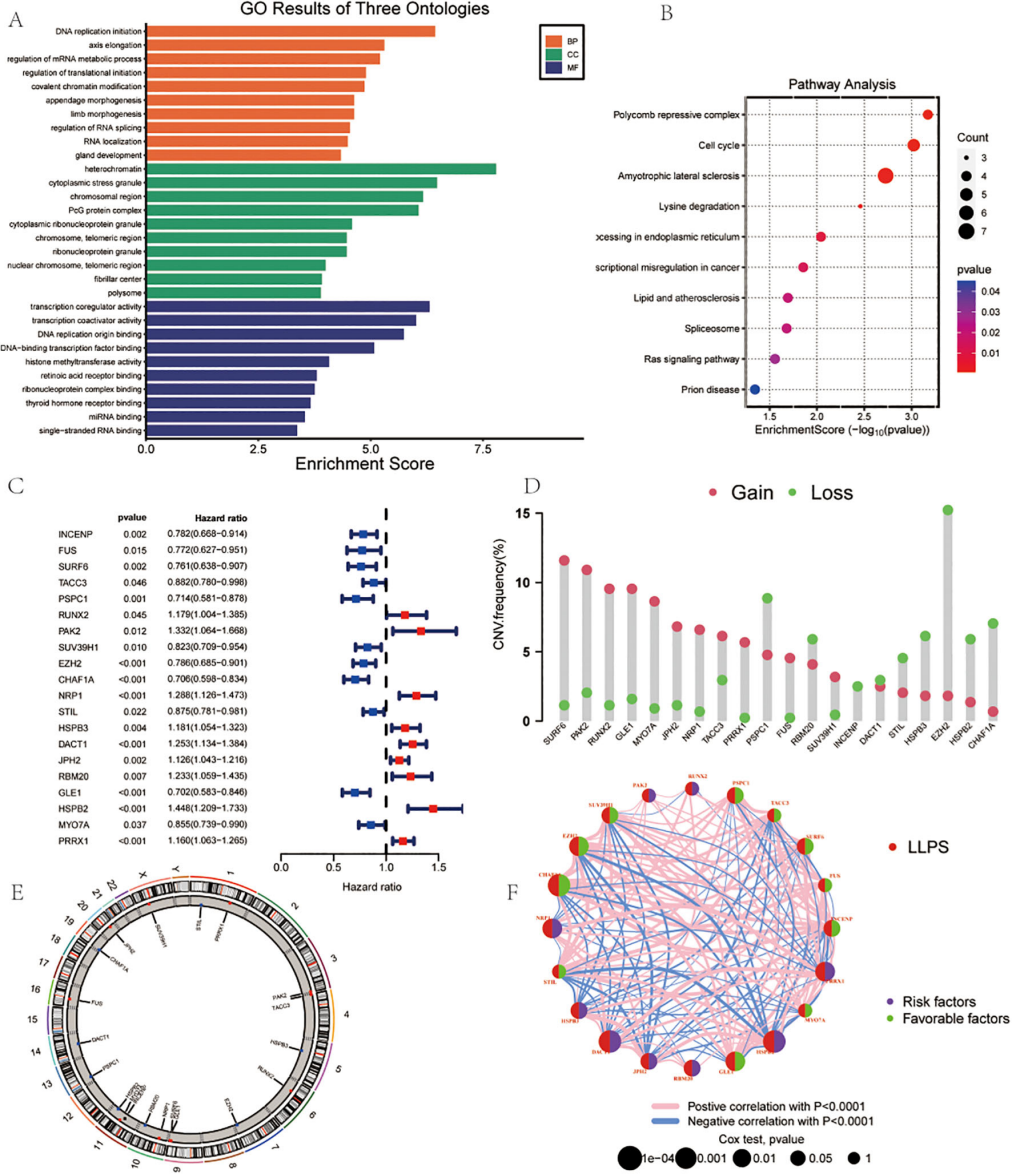
Landscape of LLPS-related Genes in Gastric Cancer. **(A)** GO Analysis. **(B)** Pathway Analysis. **(C)** Univariate COX regression analysis of the hazard ratio between 20 LLPS genes. **(D)** Frequency of CNVs in LLPS genes.**(E)** The location of CNV alteration of 14 model genes on 23 chromosomes. **(F)** Correlation network of the 20 LLPS genes.

The diagram of research was demonstrated in **Figure 1**. Using TCGA data and the “limma” package, we analyzed LLPS-related gene expression and found significant differences between two groups (**Supplementary Figure 1A**). Among them, 68 genes were upregulated and 5 were downregulated (**Supplementary Figure 1B**). Functional enrichment indicated that these genes are involved in DNA replication initiation, cell division, RNA metabolism, nuclear structure, chromosome regulation, transcription regulation, and epigenetic modifications (**Figure 2A**). Pathway analysis further highlighted enrichment in the Polycomb Repressive Complex, cell cycle, and lysine metabolism pathways (**Figure 2B**).

Univariate Cox regression analysis demonstrated the roles of different genes in survival outcomes (**Figure 2C**).To further understand the genomic alterations and interactions of these differentially expressed LLPS-related genes, we examined CNVs of 22 differentially expressed LLPS-related genes in GC, identifying chromosomal alterations and their locations. SURF6 showed the highest CNV gain (~15%), while EZH2 exhibited the highest CNV loss (~15%) (**Figure 2D**). The chromosomal distribution of CNV alterations for these genes was delineated (**Figure 2E**). A correlation network revealed positive (red lines) and negative (blue lines) correlations among the LLPS genes (**Figure 2F**).

### 3.2 Identification of LLPS clusters in GC and prognostic significance

**Figure 3:**
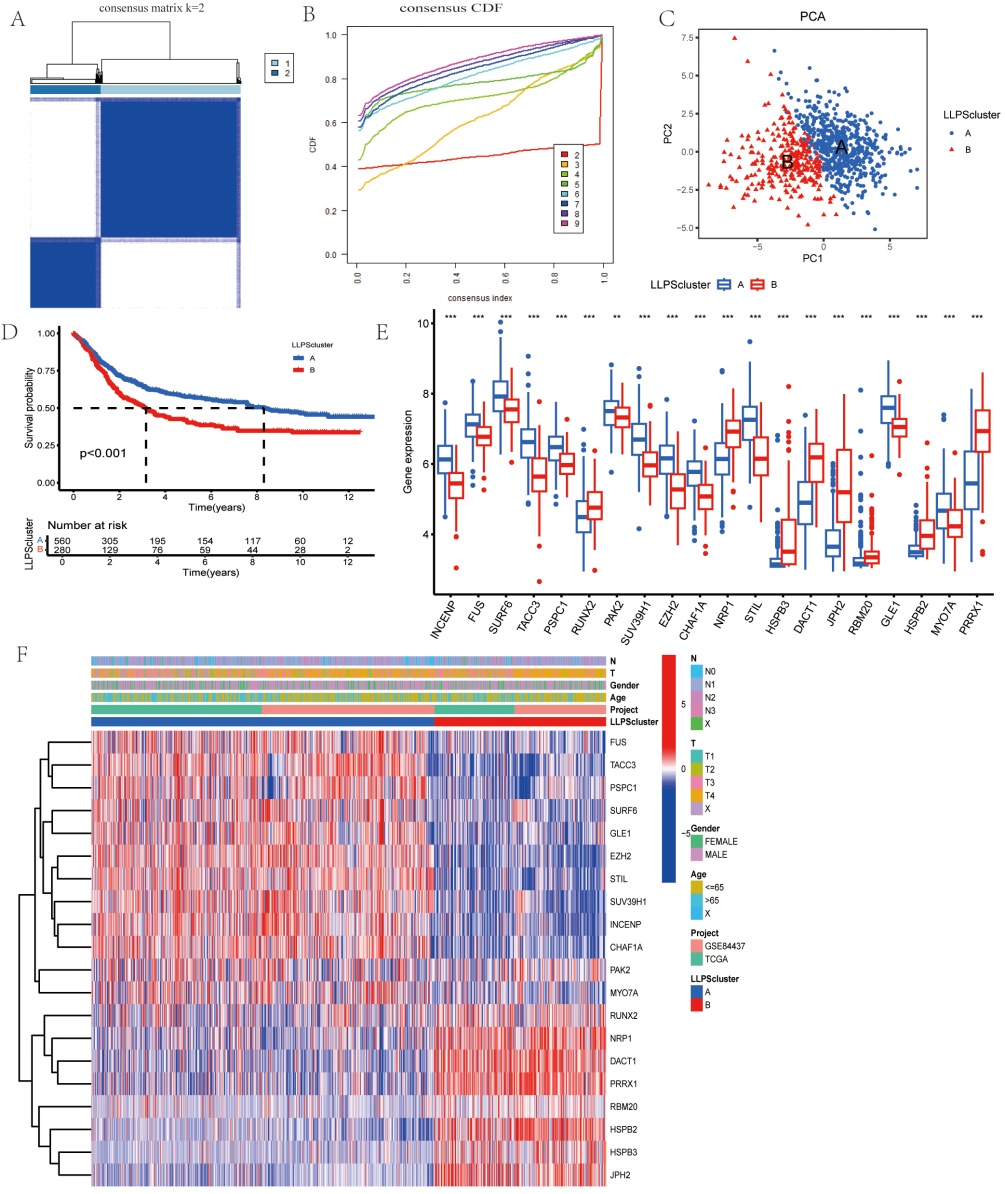
Identification of LLPS-related Gene Clusters in Gastric Cancer. **(A)** Consensus matrix showing the similarity, with k=2 indicating the division into two clusters. **(B)** Consensus cumulative distribution function (CDF) illustrating various cluster numbers (k values) to aid in determining the optimal number of clusters. **(C)**PCA of the two clusters. **(D)** Comparison of OS between the two clusters. **(E)** Gene expression boxplot comparing the expression levels of various genes between the two clusters. **(F)** Heatmap for the correlation between clinicopathologic features and the two clusters. *P<0.05, **P<0.01, ***P<0.001.

To further explore the transcriptional profiles of LLPS-related genes involved in gastric cancer tumorigenesis, we combined GC datasets from both the TCGA database and GSE84437, creating a merged TCGA-GSE cohort (N = 845). Using the “ConsensusClusterPlus” package in R, we performed an unsupervised clustering analysis, with k = 2 as the optimal number of clusters based on empirical CDF plots. This selection showed the highest intra-cluster similarity and the greatest inter-cluster separation (**Figures 3A, B**). The resulting clusters displayed two distinct expression patterns of LLPS-related genes. Additionally, cases of GC in the TCGA-GEO cohort were effectively stratified into separate groups (**Figure 3C**). Kaplan-Meier survival curves were generated to assess the prognostic value of these clusters, revealing significantly worse OS in patients within cluster B (**Figure 3D**). Univariate analysis also identified differences in gene expression between the two clusters (**Figure 3E**). Finally, we examined the clinical and pathological features of the two groups to evaluate their association with LLPS-linked gene levels (**Figure 3F**).

Next, we systematically evaluated a gastric cancer staging prediction model based on LLPS-related genes by multiple machine learning and deep learning methods. Four independent gastric cancer gene expression datasets (GSE26253, GSE27342, GSE84433, GSE26899) were integrated in this study, containing a total of 985 gastric cancer samples. To ensure the consistency and comparability of the analysis, we uniformly classified all samples into three groups of early, intermediate and advanced stages according to the AJCC/UICC TNM staging system. The specific classification criteria were as follows: both the GSE26253 dataset (n=360) and the GSE27342 dataset (n=160) directly provided the AJCC clinical staging information, and we categorized stages IB and II as the early group, stages IIIA and IIIB as the intermediate group, and stage IV as the advanced group. Some of the stage III samples in the GSE27342 dataset that were not subdivided into substages were also uniformly categorized into the intermediate group.For the GSE84433 dataset (n=357), which provides detailed TNM staging information, we grouped samples according to the combination of depth of primary tumor invasion (T), lymph node metastasis (N), and distant metastasis (M): samples that were T1-T2 and N0-N1 were categorized as the early stage group, samples that were T3-T4 or N2-N3 (without distant metastasis) were categorized as the intermediate stage group, and samples that had anysamples with distant metastases (M1) were categorized as the late group.This classification method is in line with the basic principle of AJCC staging, which takes into account the degree of local invasion, regional lymph node metastasis, and distant metastasis of the tumor. The GSE26899 dataset (n=108) had been preclassified into two groups, early (stage 1-2) and late (stage 3-4), according to the AJCC staging system, and we directly adopted its original grouping. Finally, based on the expression data of four gene markers (DACT1, EZH2, PAK2, PSPC1), we constructed a staging prediction model for 953 gastric cancer samples (319 early, 497 intermediate, and 137 advanced).

The results show that although deep learning and complex feature engineering demonstrate potential in certain aspects, the relatively simple three-gene combination (DACT1+EZH2+PSPC1) combined with the random forest model still achieves the best prediction performance (64.3% accuracy). The Bayesian approach, although slightly less accurate overall, excels in high-confidence prediction, providing an important capability for quantifying uncertainty in clinical applications.

Based on these findings, we further explored the expression patterns and functional significance of LLPS-related genes in gastric cancer. As shown in **Supplementary Data 4-9**, the differential expression patterns of key LLPS genes in different gastric cancer stages were confirmed by integrative analysis, validating their potential value as staging prediction biomarkers.

### 3.3 Tumor−microenvironment features correlated with LLPS clusters

**Figure 4:**
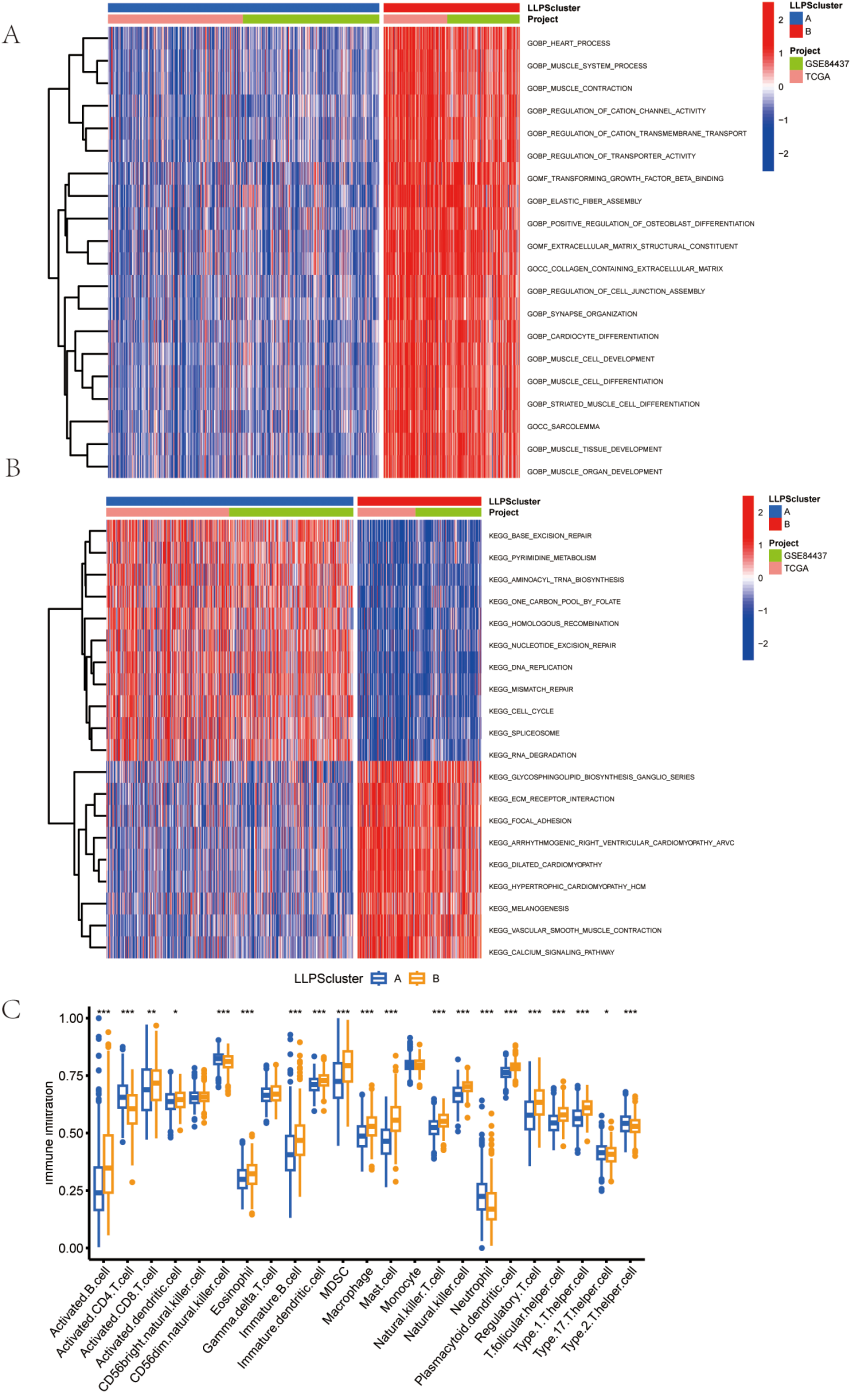
Features of the Tumor Microenvironment (TME) in the LLPS Clusters Identified in Gastric Cancer. **(A)** Comparison of the GSVA of Go Gene Ontology (GO) Terms between the two LLPS clusters in GC. **(B)** Comparison of the GSVA of biological pathways between the two LLPS clusters in GC. **(C)**Abundance of 23 infiltrating immune cell types in the two LLPS clusters.

In order to rigorously investigate the contributions of LLPS-related genes to the gastric tumoral milieu, we implemented GSVA analysis. As illustrated in **Figure 4A, a** substantial enrichment of Cluster B was observed in multiple biological pathways, including muscle contraction, cation channel activity, and transporter activity regulation. In the KEGG Pathway analysis showed that Cluster A and Cluster B exhibited significant results. differences in gene expression. Genes in Cluster A were enriched in pathways connected to cell proliferation and DNA repair, including the “cell cycle” and “genome repair”, while genes in Cluster B were enriched in pathways associated with cellular homeostasis, apoptosis, and other metabolic processes (**Figure 4B**). Moreover, using ssGSEA, Cluster B exhibited higher levels of myeloid-derived suppressor cells (MDSCs), activated B cell, regulatory T cells (Tregs), activated CD8 T cell, and macrophages compared to Cluster A (**Figure 4C**).

### 3.4 Construction and validation of the LLPS signature and associated prognostic scoring system

**Figure 5:**
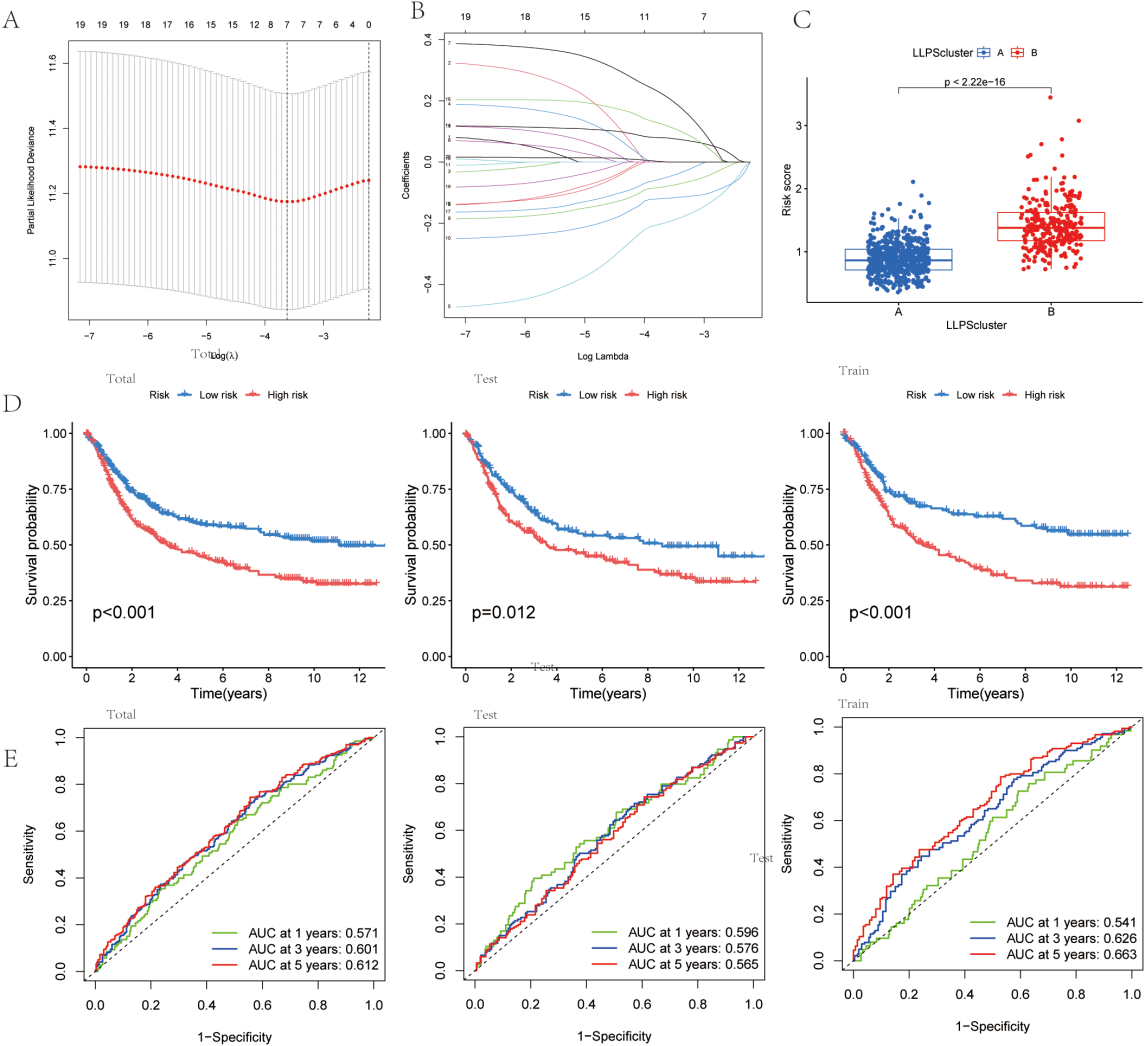
Identification of an LLPS-related Differentially Expressed Gene **(DEG)** Signature and Risk Model for Gastric Cancer. **(A)** LASSO regression for 7 candidate genes. **(B)** Cross-validation for 7 OS-related genes in the LASSO regression.**(C)** Risk score in the LLPS clusters.**(D)**Kaplan-Meier curve analysis for OS in total, test,and train Cohort. **(E)** The ROC curve analysis demonstrated the predictive efficiency of the prognostic score in total, test,and train Cohort.

To explore the molecular basis of GC progression, we selected prognostic subtype-associated genes identified by Lasso-based Cox regression (**Figures 5A, B**). The risk index was created from four gene signatures linked to prognostic subtypes. This prognostic score was calculated using expression profiles of these genes, as explained below:

Prognostic score=0.140×DACT1 + 0.384×PAK2-0.212×EZH2-0.307×PSPC1

Risk score distribution across LLPS clusters was visualized (**Figure 5C**). The alluvial diagram showed how gastric cancer patients were allocated between the two LLPS clusters and the two prognostic-score groups (**Supplementary Figure 2A**). Survival analysis with the Kaplan-Meier method demonstrated significantly worse outcomes for patients in the high prognostic score group compared to those in the low group (**Figure 5D**). Additionally, the predictive ability of the LLPS-related differentially expressed gene signature was assessed through time-dependent ROC curve analysis, showing strong prognostic accuracy at 1, 3, and 5 years (**Figure 5E**).

**Figure 6:**
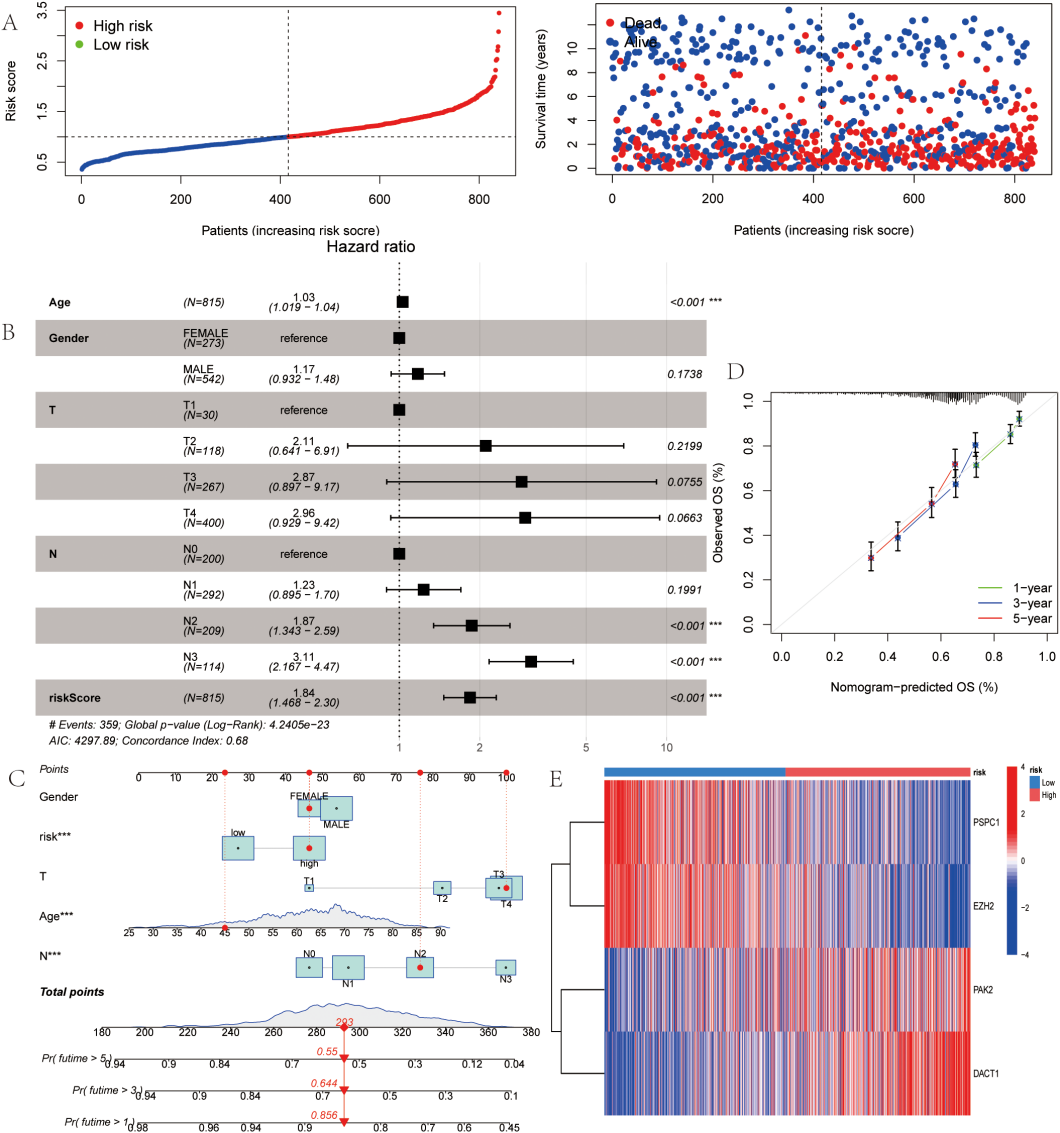
LLPS-Associated Risk Scoring System for Gastric Cancer. **(A)** Ranked dot of prognostic score distribution and patient survival status. **(B)** Multivariate independ- ent prgnostic analysis. **(C)** Nomogram was developed by integrating gender age, TNM stage, and LLPS risk. **(D)** Calibration plots to assess the accuracy of nomogram. **(E)** The Heatmap of the expression of the four OS-related genes. *P<0.05, **P<0.01, ***P<0.001.

**Figure 7:**
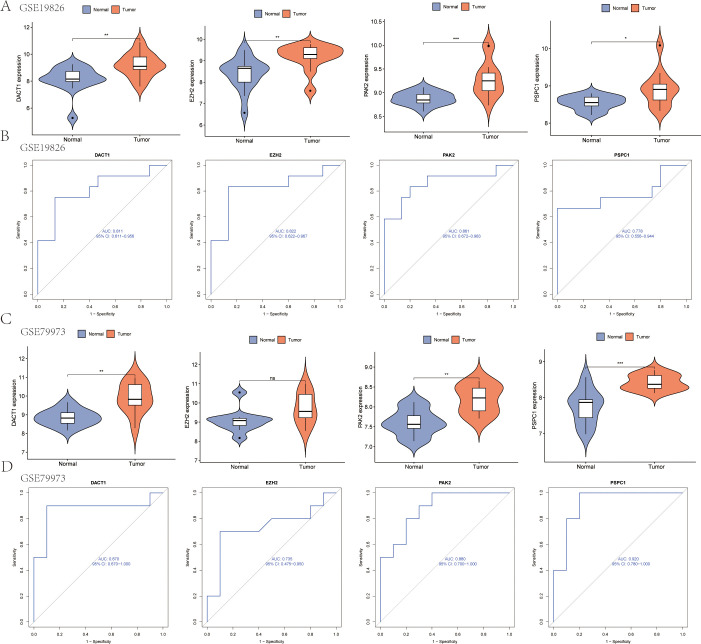
Gene Expression Analysis and ROC Curve Comparison in test sets (GSE19826 and GSE79973). **(A)** Expression analysis of the four genes in the GSE19826 data set. **(B)** ROC curve analysis of the four genes in the GSE19826 data set. **(C)** Expression analysis of the four genes in the GSE79973 data set. **(D)** ROC curve analysis of the four genes in the GSE79973 data set. *P<0.05, **P<0.01, ***P<0.001. Ns, Not Significant.

Multivariable stratification of the high-risk group indicated a significant increase in mortality risk, as shown in the survival distribution plot (**Figure 6A**). In the multivariate Cox regression analysis, LRRS was associated with a hazard ratio of 1.84 (95% CI: 1.468–2.30, *P*<0.001; **Figure 6B**). The nomogram combined multiple variables, with the risk group serving as an important predictive factor (**Figures 6C, D**). The heat map revealed distinct expression patterns of the four genes, consistent with the prognostic score (**Figure 6E**). Moreover, the genes identified earlier were validated in two independent test sets (GSE19826 and GSE79973). The results showed high expression levels of DACT1, PAK2, PSPC1, and EZH2 in GC (**Figures 7A, C**). The ROC analysis confirmed the predictive power of these genes (**Figures 7B, D**).

### 3.5 Comprehensive evaluation of immunological activity and tumor mutational burden across distinct prognostic score categories

**Figure 8:**
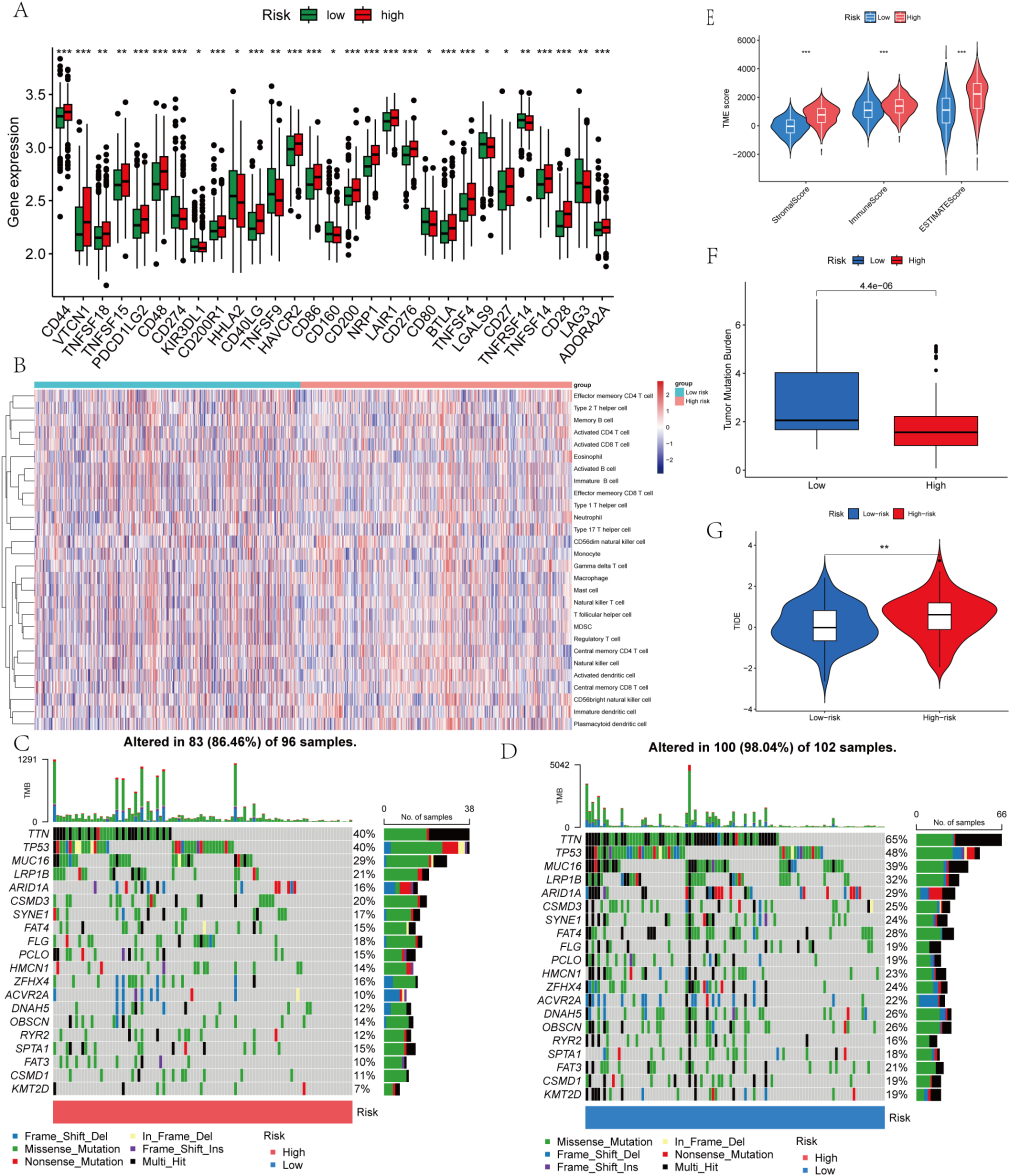
Immune Microenvironment and Tumor Mutational Burden (TMB) of Gastric Cancer Tissues with Different LLPS-Associated Prognostic Scores. **(A)** Expression levels of immune checkpoint genes of the two risk groups. **(B)** Correlation between LRRS and immune checkpoint genes. **(C)** The mutation frequency of the top 20 genes in the low LRRS group. **(D)** The mutation frequency of the top 20 genes in the high LRRS group. **(E)** TME score of the two risk groups. **(F)** TMB of the two risk groups. **(G)** TIDE of the two risk groups. *P<0.05, **P<0.01, ***P<0.001.

**Figure 9:**
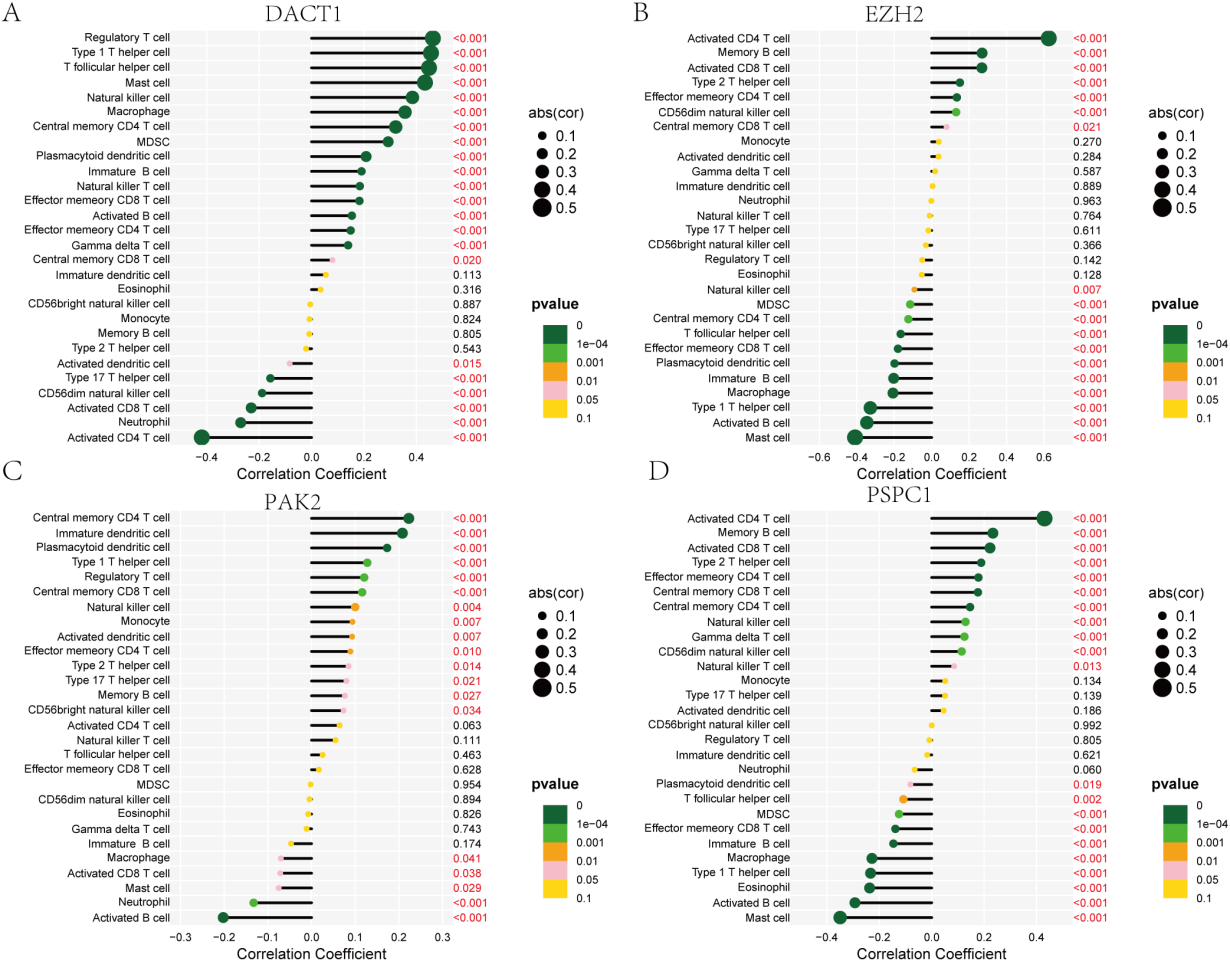
Correlation Analysis of DACT1, EZH2, PAK2, and PSPC1 with Immune Cell Types. **(A)** DACT1 with each type of infiltrating immune cell. **(B)** EZH2 with each type of infiltrating immune cell. **(C)** PAK2 with each type of infiltrating immune cell. **(D)** PSPC1with each type of infiltrating immune cell.

Cancer progression and immunotherapy response are heavily influenced by the immune microenvironment. Consequently, our research aimed to analyze the tumor microenvironment pattern among individuals with GC grouped into high- and low-risk categories. We evaluated differences in the immunophenotypic score. The low-risk group demonstrated an elevated immunophenotypic score, suggesting a more promising immunotherapeutic response potential (**Figure 8A**). The high-risk score group exhibited a strong positive correlation with the inhibitory immune checkpoints HAVCR2 and PDCD1 (**Supplementary Figure 3A**). The immune cell subpopulation correlation analysis indicated that prototypical immunosuppressive cells, including Tregs, MDSCs, and macrophages, are co-enriched within the high-risk score group, thereby further weakening the antitumor functions of effector T cells and NK cells (**Supplementary Figure 3B**). The heatmap displayed the distinctions between the two groups of immune cells (**Figure 8B**). Subsequent mutational profiling of the 20 most frequently altered genes demonstrated a significantly elevated mutational frequency in the low-risk group (**Figures 8C, D**). By analyzing the gene expression landscape, we determined stromal and immune scores for both cohorts (**Figure 8E**). A strong positive correlation was observed between the prognostic score and TMB, with significantly higher values in the low-risk cohort relative to the high-risk cohort (**Figure 8F**). Additionally, we observed that the TIDE score in the high-risk group was notably higher (**Figure 8G**). DACT1 showed strong positive correlations with Tregs, T helper cells (Th1 and Tfh). Conversely, it showed negative correlations with activated CD4 T cells and CD8 T cells (**Figure 9A**). EZH2 demonstrated significant positive correlations with activated CD4 T cells, memory B cells, and activated CD8 T cells, while showing negative relationships with monocytes and certain innate immune cells (**Figure 9B**). PAK2 exhibited prominent positive correlations with central memory CD4 T cells, immature dendritic cells, and plasmacytoid dendritic cells, while showing weak or even negative correlations with activated B cells and mast cells (**Figure 9C**). PSPC1 showed strong positive correlations with adaptive immune components such as activated CD4 T cells, memory B cells, and activated CD8 T cells, but demonstrates negative correlations with certain myeloid immune cells (such as MDSCs) (**Figure 9D**).

### 3.6 Gene Expression and CRISPR Functional Dependency

**Figure 10:**
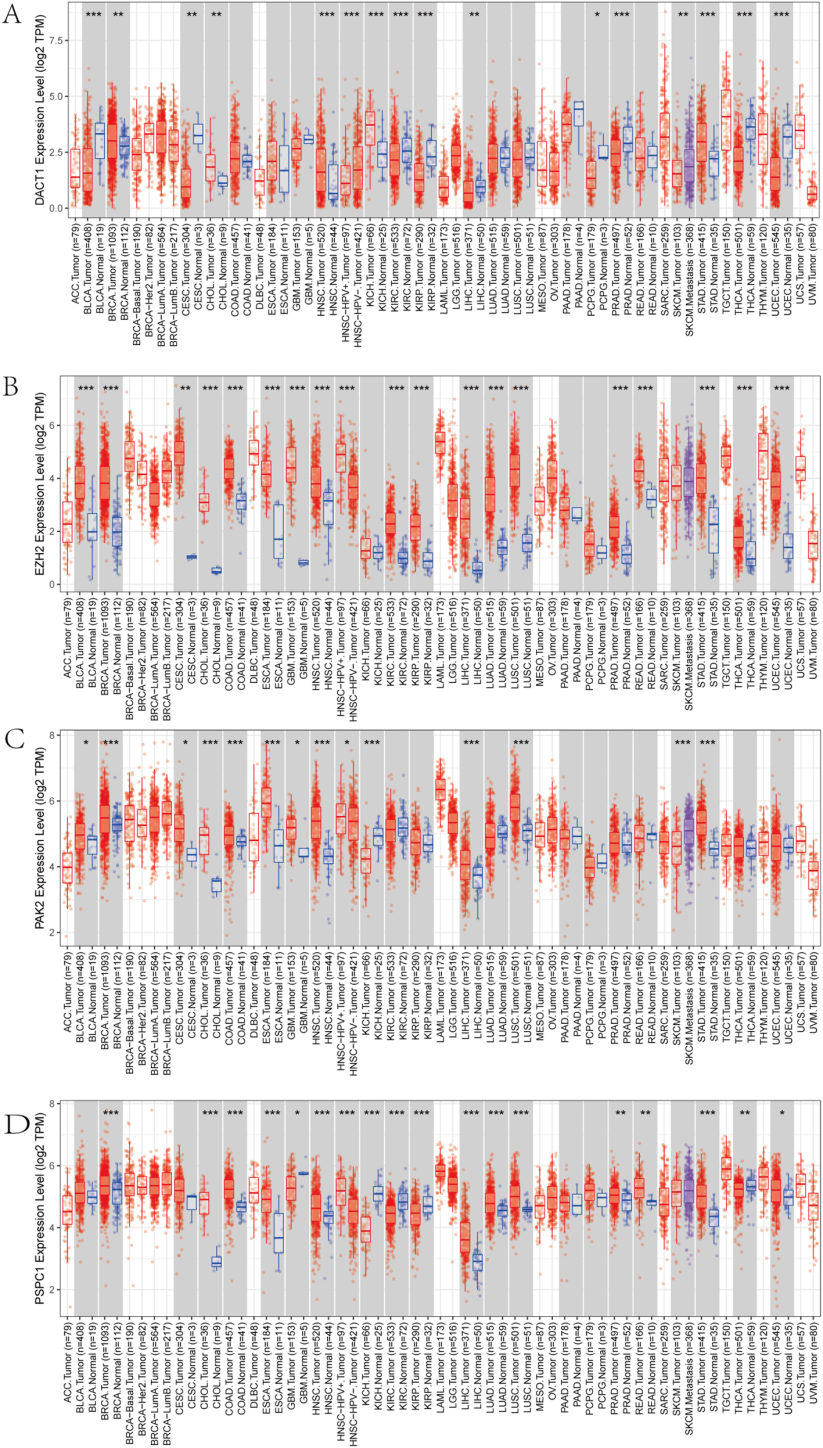
**(A-D)** Pan-cancer Analysis of Gene Expression Differences: DACT1, EZH2, PAK2, and PSPC1. *P<0.05, **P<0.01, ***P<0.001.

**Figure 11:**
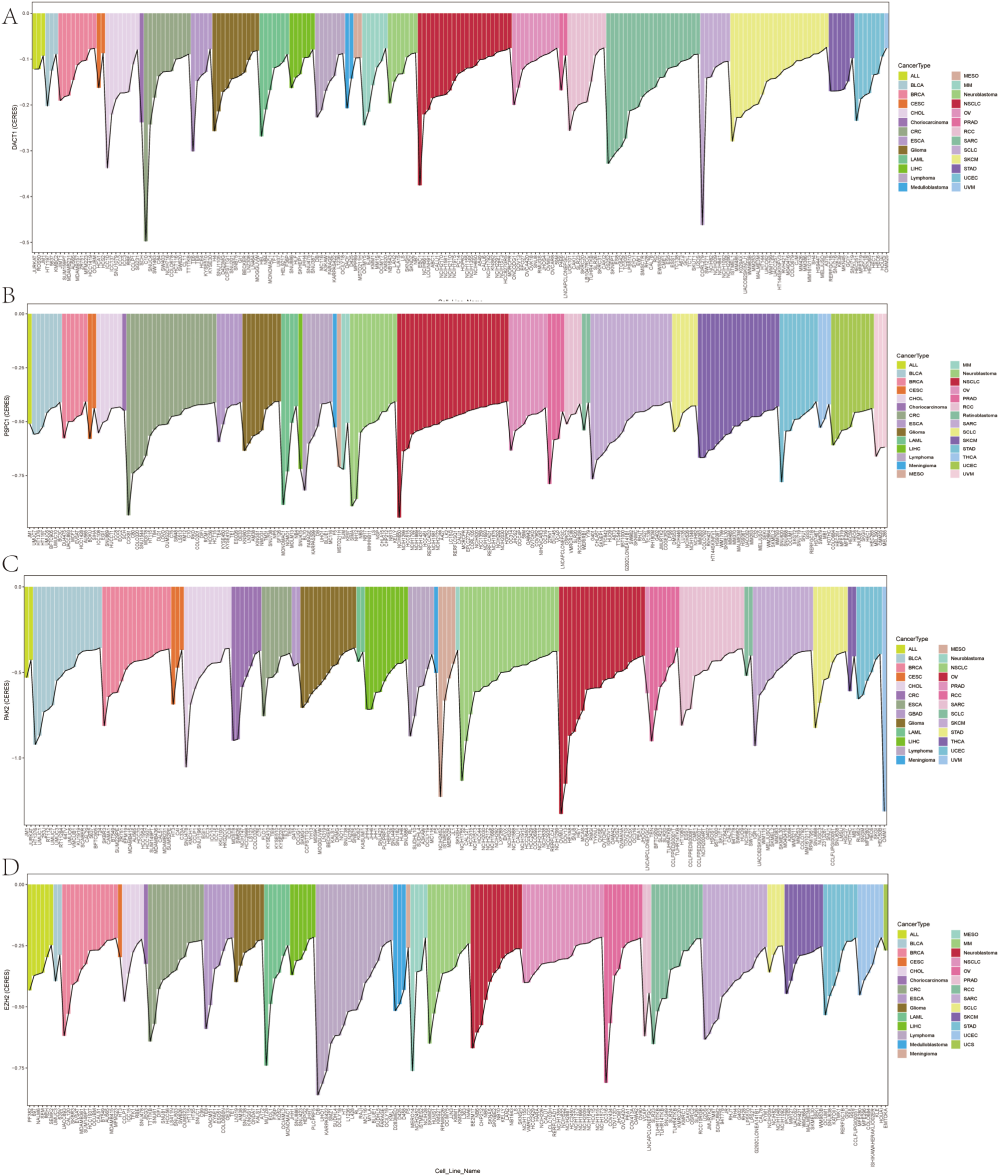
**(A-D)** Genome-Wide CRISPR Screening Dependency Scores in Gastric Cancer Using CERES Algorithm.

**Figure 12:**
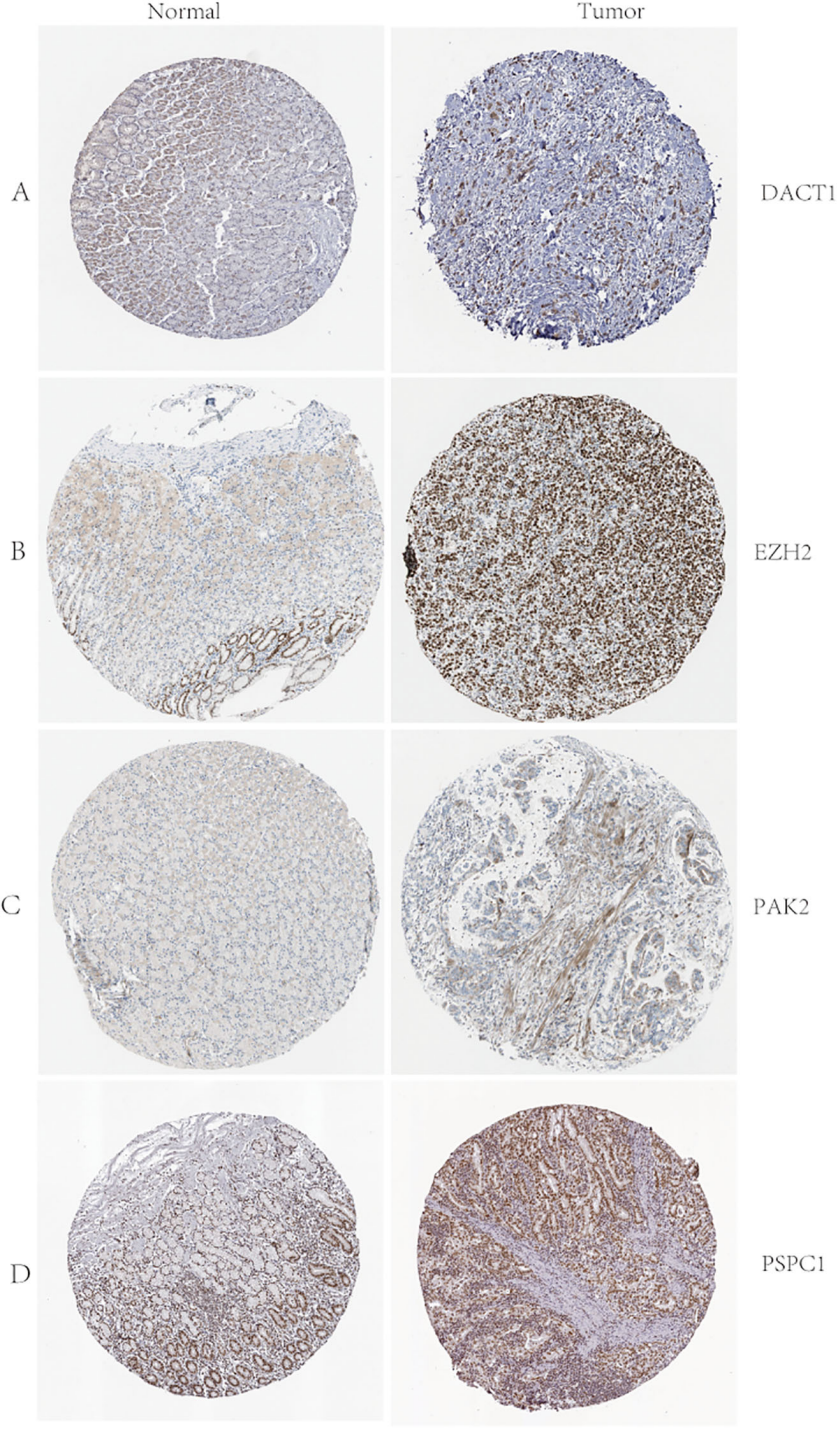
**(A-D)** D ACT1, EZH2, PAK2, and PSPC1 Protein Expression in Normal and Tumor Tissues: Immunohistochemistry Analysis from the Human Protein Atlas.

We conducted multi-dimensional analyses focused on four candidate genes-*DACT1, EZH2, PAK2, and PSPC1*. **Figure 10** showed the expression levels of these four genes in malignant (red) and normal (blue) tissues across multiple cancer types, including BRCA, COAD, LUAD, and KIRC. EZH2 and PSPC1 were significantly upregulated in the majority of cancers. DACT1 was notably downregulated in several cancer types. PAK2 exhibited cancer-type specificity: it was distinctly elevated in some cancers while showing no significant difference in others. **Figure 11** presented dependency scores from the CERES algorithm-based genome-wide CRISPR knockout data in GC cell lines, indicating that these four genes are important for cancer survival. We used immunohistochemistry (IHC) slides from the Human Protein Atlas database to compare the protein abundance and localization of DACT1, EZH2, PAK2, and PSPC1 in normal and corresponding tumor tissues. DACT1 showed weak to moderate immunoreactivity in normal tissues and no significant change in tumor tissues (**Figure 12A**). In contrast, EZH2 displayed stronger, more widespread brownish staining in tumor tissues, suggesting overall upregulation in cancer cells (**Figure 12B**). PAK2 appeared to be expressed at moderate-to-low levels in normal tissues but showed partial elevation in tumor tissues, indicating its potential role in tumorigenesis (**Figure 12C**). PSPC1 demonstrated prominent staining in tumor tissues, with some regions showing strong positivity (**Figure 12D**).

### 3.7 Assessment of anticancer treatment efficacy in cohorts stratified by high versus low prognostic scores

**Figure 13:**
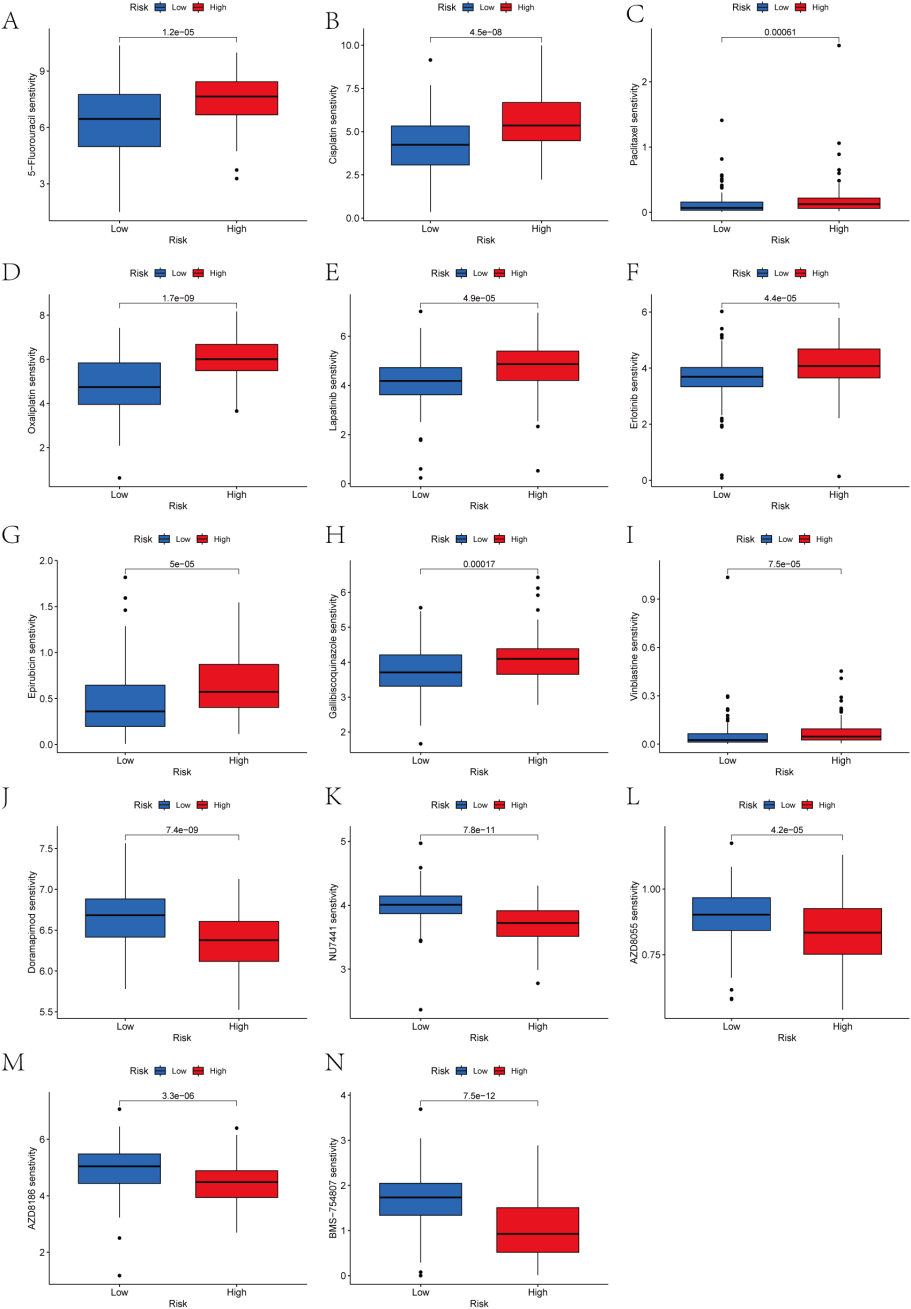
**(A-N)** Drug sensitivity analysis in gastric cancer: risk group comparison.

We analyzed the drug responsiveness of groups with high or low prognostic scores to various chemotherapeutic and targeted agents (**Figure 13**). Boxplots clearly showed that the low score group exhibited heightened sensitivity to 5-Fluorouracil, Cisplatin, Paclitaxel, Oxaliplatin, Lapatinib, Erlotinib, Epirubicin, Galliblocquinazole, and Vinblastine compared with the high prognostic score group (*P*<0.001). Conversely, the high prognostic score cohort demonstrated increased sensitivity to Doramapimod, NU7441, AZD8055, AZD8186, and BMS-754807 (*P*<0.001).

### 3.8 Expression levels of LLPS genes expression in GC cell lines

**Figure 14:**
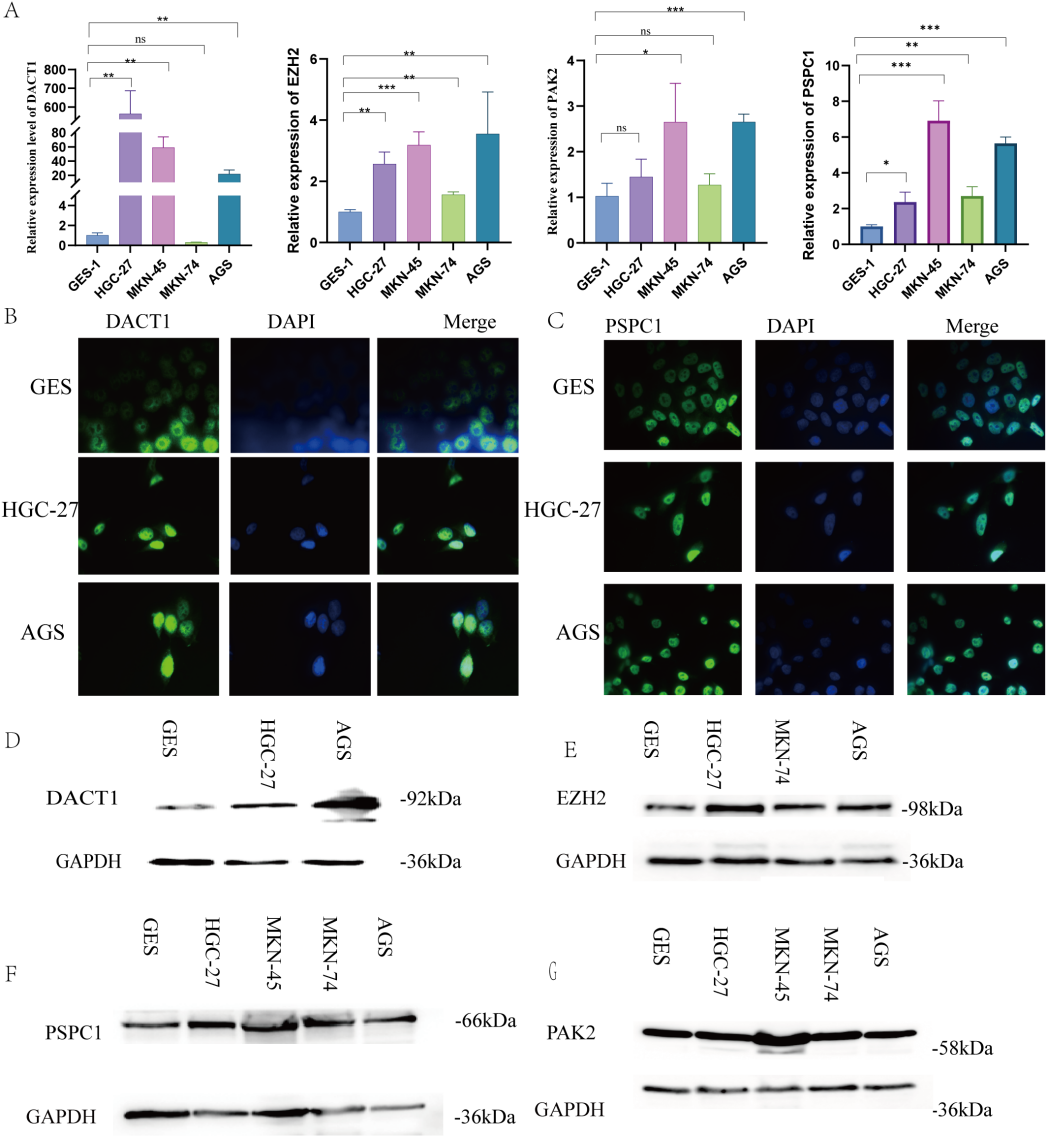
Verification Expression Levels of LLPS Genes Expression in GC Cell Lines. **(A)** The relative expression levels of DACT1, EZH2, PSPC, and PAK2 mRNA. **(B)** Immunofluorescence of DACT1. **(C)** Immunofluorescence of PSPC1. **(D-F)** Western Blot of DACT1, EZH2, PAK2 and PSPC1.**P < 0.01, ***P < 0.001. Ns:Not Significant.

As described earlier, this novel index was based on four LLPS genes (including DACT1, EZH2, PAK2, and PSPC1). Therefore, we next performed qRT-PCR to determine the mRNA expression levels of these target genes in GES, HGC-27, MKN-45, MKN-74, and AGS cell lines (**Figure 14A**). The experimental findings were similar to the results from GEO and TCGA databases. IF analysis showed the localization of DACT1 and PSPC1 (**Figure 14B, C**). The Western Blot results demonstrated high protein expression in cancer cell lines (**Figure 14D-G**). Collectively, these results support the relevance of the identified LLPS genes in gastric cancer (GC) and their potential roles in tumor biology.

### 3.9 PSPC1 plays an important role in gastric cancer cell proliferation and migration

To verify the functional role of PSPC1 in gastric cancer progression, we performed loss-of-function experiments in two gastric cancer cell lines, HGC-27 and AGS.

**Figure 15:**
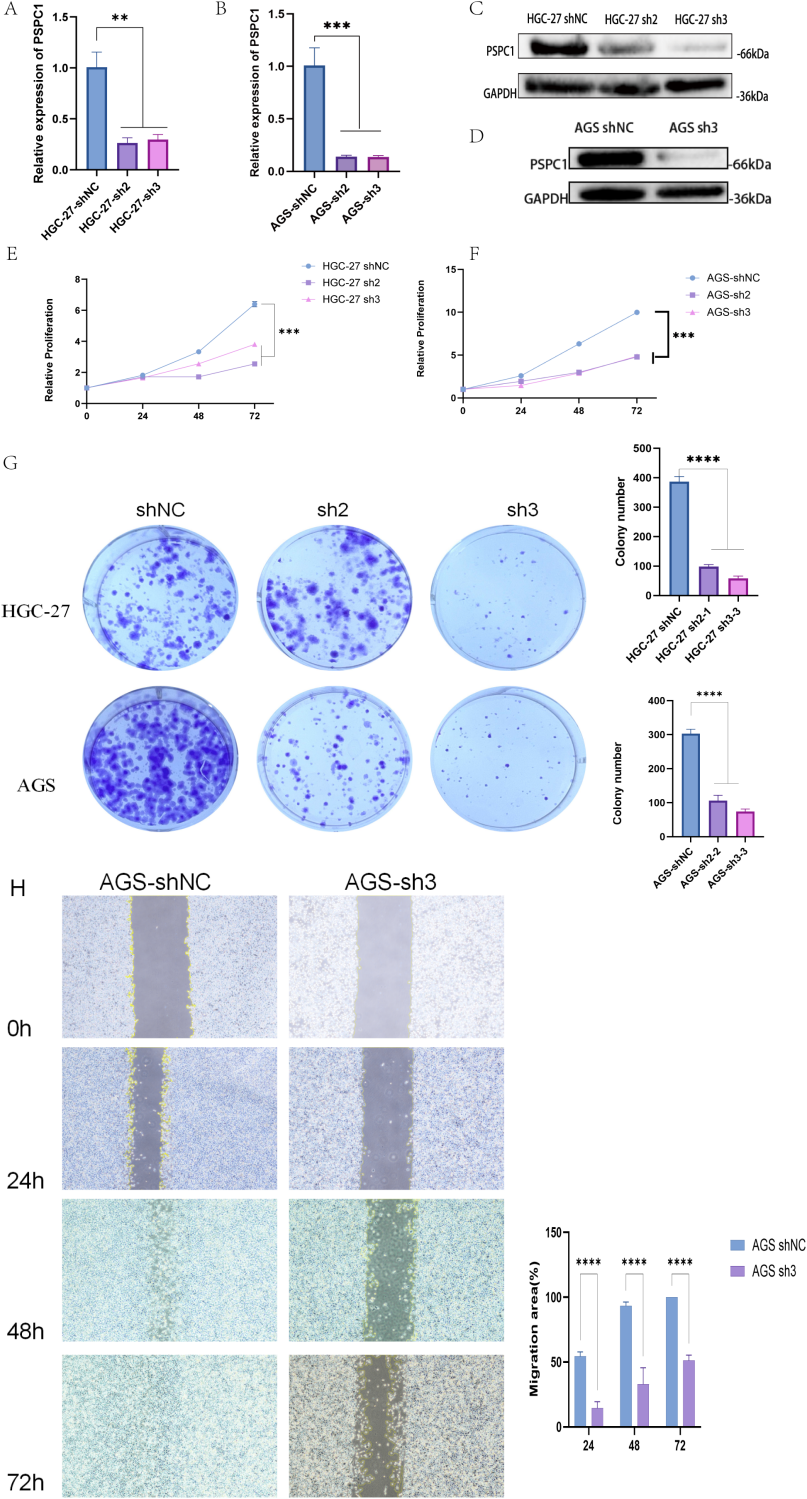
PSPC1 silencing inhibits the proliferation of GC *in vitro*. **(A)** RT-PCR verified the expression of depleted PSPC1 in the HGC-27. **(B)** RT-PCR verified the expression of depleted PSPC1 in the AGS. **(C)** Western blot showing depleted PSPC1 expression by two independent shRNA (sh2 and sh3) in HGC-27. **(D)** Western blot showing depleted PSPC1 expression by shRNA (sh3) in AGS. **(E)** Proliferation rates of PSPC1-depleted cells assessed by CCK8 assay in HGC-27. **(F)** Proliferation rates of PSPC1-depleted cells assessed by CCK8 assay in AGS. **(G)** Colony formation assay was performed on HGC-27 and AGS cells treated with PSPC1 silencing to validate the growth ability of the indicated cells *in vitro*. **(H)** Representative images and quantitative analysis of wound healing assay of AGS cells transfected with shRNA (sh3) and vector. **P < 0.01, ***P < 0.001, ****P < 0.0001.

First, we designed shRNAs (sh2 and sh3) targeting PSPC1 and established stable knockdown cell lines by lentiviral infection. RT-qPCR assay showed that in HGC-27 cells, both sh2 and sh3 significantly reduced the mRNA expression level of PSPC1 (**P < 0.01) (**Figure 15A**); in AGS cells, both shRNAs similarly effectively inhibited PSPC1 expression (***P <0.001) (**Figure 15B**). Western blot analysis further confirmed the knockdown effect at the protein level (**Figure 15C, D**). Based on the knockdown efficiency, we selected sh3 for subsequent functional experiments. To assess the effect of PSPC1 on cell proliferation, we performed CCK-8 and clone formation assays. CCK-8 results showed that PSPC1 knockdown significantly inhibited the proliferative ability of both gastric cancer cells. In HGC-27 cells, the difference in proliferation between the knockdown group and the control group reached a significant level at 48 hours of culture (***P<0.001) (**Figure 15E**); in AGS cells, this inhibition was more pronounced, with the proliferative capacity of the knockdown group decreasing to approximately 50% of that of the control group at 72 hours (***P<0.001) (**Figure 15F**). Clone formation assays further supported this finding: the number of clones formed in HGC-27 and AGS cells significantly decreased from approximately 400 and 300 to less than 100 after PSPC1 knockdown, respectively (****P<0.0001) (**Figure 15G**), suggesting that PSPC1 has an important role in the long-term proliferative capacity of gastric cancer cells.

In addition, we evaluated the effect of PSPC1 on cell migration ability by scratch healing assay. The results showed that PSPC1 knockdown significantly inhibited the migration ability of AGS cells.At each time point of 24, 48 and 72 hours, the migration area of the sh3 group was significantly lower than that of the control group (****P < 0.0001). In particular, at 72 hours, the scratches in the control group were essentially healed (100% of the migrated area), whereas the knockdown group migrated only about 50% (**Figure 15H**).

Taken together, these results indicate that PSPC1 plays an important role in promoting the proliferation and migration of gastric cancer cells, suggesting that it may act as a promoter of gastric cancer progression.

## 4 Discussion

Recent studies have shown that tumorigenesis and development are closely related to gene mutation, amplification, epigenetic abnormalities and signaling pathway imbalance (24, 25), in which LLPS plays an important role (26). In this study, the expression and mutation patterns of LLPS-related genes in gastric cancer were systematically analyzed for the first time, and gastric cancer patients were classified into two LLPS subtypes with different prognoses, clinicopathological features, and immune infiltration patterns by unsupervised clustering. A four-gene risk score model (LRRS) containing *DACT1, PAK2, EZH2, and PSPC1* was further constructed, which was significantly associated with patient survival, clinical features, and genomic alterations.

The four prognostic genes include two scaffold genes and two client genes. DACT1 showed heterogeneity in different tumors: it was downregulated in bladder (27), breast (28), and cervical (29) cancers and upregulated in colon and squamous cancers (30, 31). In the present study, DACT1 was found to be highly expressed in gastric cancer cells, suggesting its unique role in gastric cancer. PSPC1 is involved in RNA processing and transcriptional regulation, and is a key component of parafollicular plaque formation (32), which promotes the formation of intracellular LLPS structures by binding RNA (33). Although PSPC1 is associated with cell proliferation and metastasis in a variety of tumors (34–36), its specific mechanism in gastric cancer has not been previously elucidated. In the present study, we confirmed the critical role of PSPC1 in gastric cancer progression by functional experiments, which is consistent with its report of promoting malignant phenotypes in other tumors (37–41), suggesting that PSPC1 may be involved in gastric cancer development by influencing the LLPS process, providing a theoretical basis for the development of targeted therapeutic strategies against PSPC1. EZH2, as an epigenetic regulator, affects gene expression by regulating histone methylation, and promotes tumor growth, metastasis, and drug resistance in a variety of malignancies (42, 43).In this study, we confirmed that EZH2 is highly expressed in gastric cancer, and CERES algorithm analysis showed that several gastric cancer cell lines were highly dependent on EZH2, supporting its potential as a therapeutic target. PAK2 is involved in cytoskeletal remodeling, migration, and cell cycle regulation, and in lung squamous carcinoma, it promotes proliferation and invasion (44).We found that PAK2 was generally upregulated in gastric cancer tissues and highly dependent on it in certain cell lines, consistent with its critical role in maintaining tumor cell function.

Tumorigenesis is affected by both genetic mutations and immune dysregulation (45, 46).The high LRRS group showed a complex immune profile: increased immune cell infiltration, elevated stromal scores, immunity scores, and ESTIMATE scores, but greater immunosuppression. Genomic analysis revealed that TTN mutations induced CD8+ and CD4+ T cell infiltration (47); TP53 mutations affected cell cycle and DNA repair and remodeled the immune microenvironment (48); MUC16 mutations increased neoantigen production but may inhibit NK cell killing (49, 50); and ARID1A mutations regulated the tumor inflammatory microenvironment and may enhance immunotherapy sensitivity (51). Low-risk groups may have more “benign” mutations, and high mutation loads enhance tumor antigenicity, promote immune recognition, and improve prognosis.

Drug sensitivity analysis revealed therapeutic strategies for different prognostic groups.The low-scoring group was more sensitive to first-line chemotherapeutic agents such as 5-fluorouracil, cisplatin, paclitaxel, oxaliplatin, and epirubicin (p<0.001) (52), as well as responded well to HER2/EGFR-targeted agents such as lapatinib and erlotinib, which was consistent with the results of clinical trials in HER2-positive or EGFR-highly-expressed gastric cancer (53, 54). The high-scoring group, on the other hand, was more sensitive to novel kinase inhibitors such as Doramapimod (p38 MAPK inhibitor), NU7441 (DNA-PK inhibitor), and AZD8055 (mTOR inhibitor) (55–57), which provides a rationale for individualized treatment.

In this study, LLPS gene was firstly used as a prognostic marker for gastric cancer, and its biological mechanism, immune characteristics and mutation spectrum were systematically explored, which provided a new idea for clinical individualized treatment.However, the study still has limitations: clinical samples are needed for further validation; the specific mechanisms of the four risk genes in LLPS and their interrelationships need to be explored in depth.Nevertheless, this study provides important candidate molecules for prognostic assessment and therapeutic target development in gastric cancer.

## 5 Conclusion

In conclusion, our research identified 20 genes related to LLPS that are linked to the prognosis of GC patients. By utilizing these genes, we effectively categorized patients into two distinct subtypes, which have different pathway activity, prognosis, clinicopathological features and immune cell infiltration. In addition,we created a prognostic model based on four of LLPS genes. Our results indicate that integrating scores based on LLPS genes applied in clinical practice could serve as a valuable instrument for predicting GC prognosis.

## Data Availability

The datasets presented in this study can be found in online repositories. The names of the repository/repositories and accession number(s) can be found in the article.
