# Supplementary material for: Liquid-liquid phase separation in gastric cancer: identifying novel biomarkers and therapeutic targets through gene signature analysis
Source: Front Immunol. 2025 Sep 1;16:1620390. doi: 10.3389/fimmu.2025.1620390 (PMC12434088; doi:10.3389/fimmu.2025.1620390)
Supplement: Supplementary file 1 [file DataSheet1.docx]

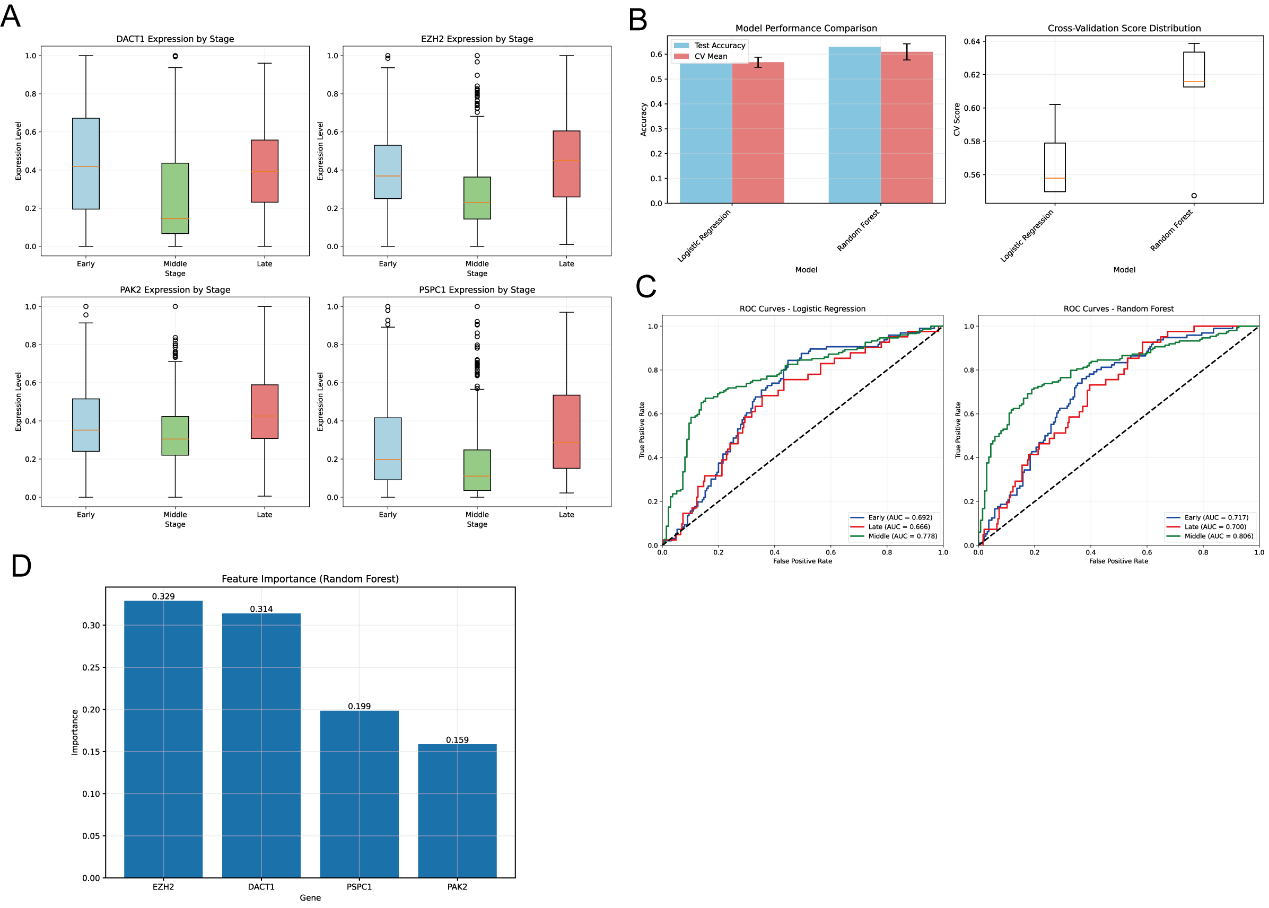


**Supplementary Figure 4 Construction and evaluation of gastric cancer stage prediction model based on four-gene expression** (A) Expression levels of four genes (DACT1, EZH2, PAK2, PSPC1) in different stages of gastric cancer. (B) Performance comparison and cross-validation score distribution of random forest and logistic regression models. (C) ROC curves of logistic regression (left) and random forest (right) models demonstrating the classification performance of early, intermediate and advanced stages. (D) Characteristic importance scores of the four genes in the random forest model.


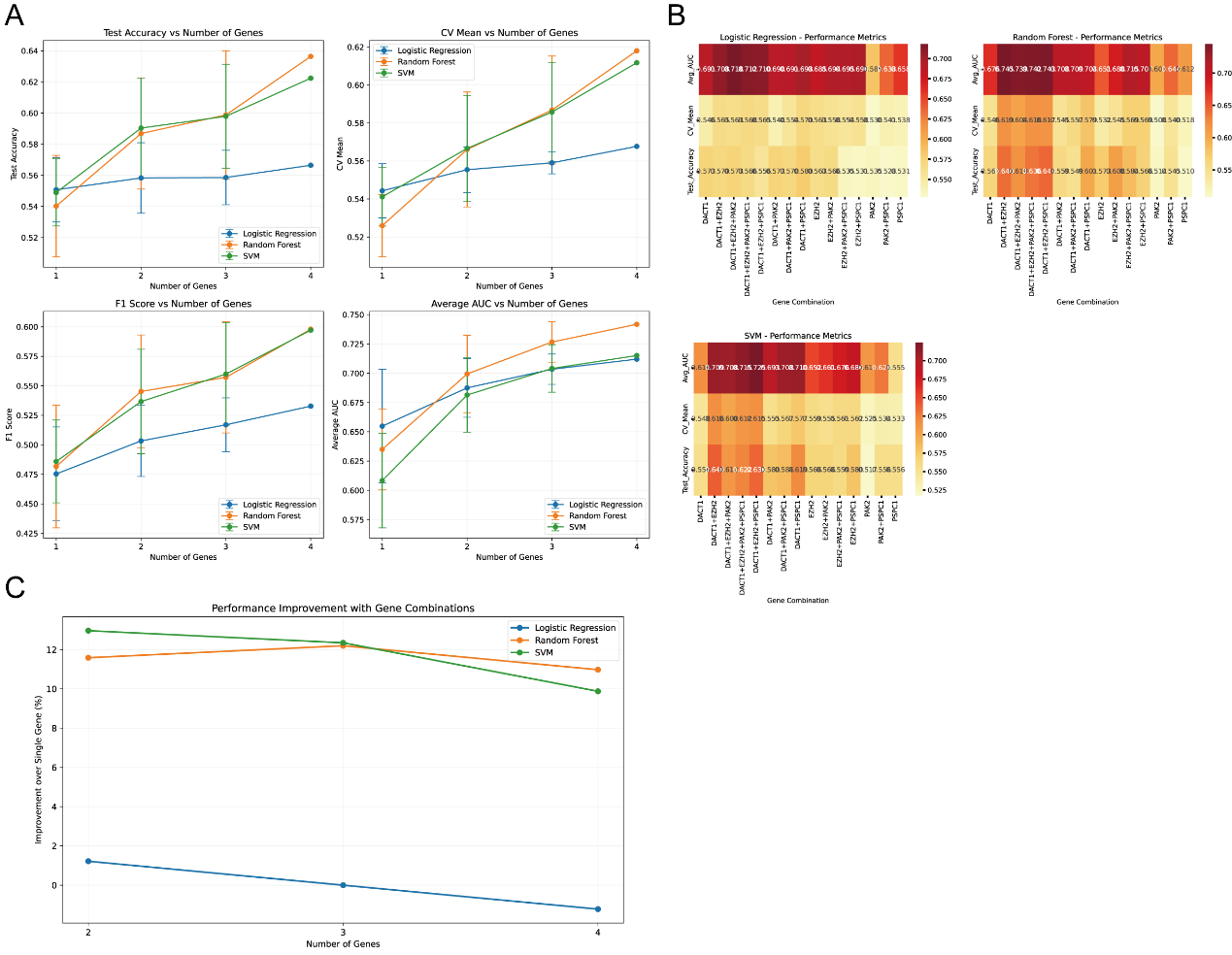


**Supplementary Figure 5 Effects of different gene combinations on the prediction performance of gastric cancer staging** (A) Relationship between the number of genes and model performance. The test accuracies, CV means, F1 scores, and mean AUC values of the three models, logistic regression, random forest, and SVM, when using different numbers of genes (1-4) are shown. (B) Heatmap of the performance of the three models with different combinations of genes. The top row is logistic regression, the middle row is random forest, and the bottom row is SVM model. (C) Trend of the performance of the three models as the number of genes increases.


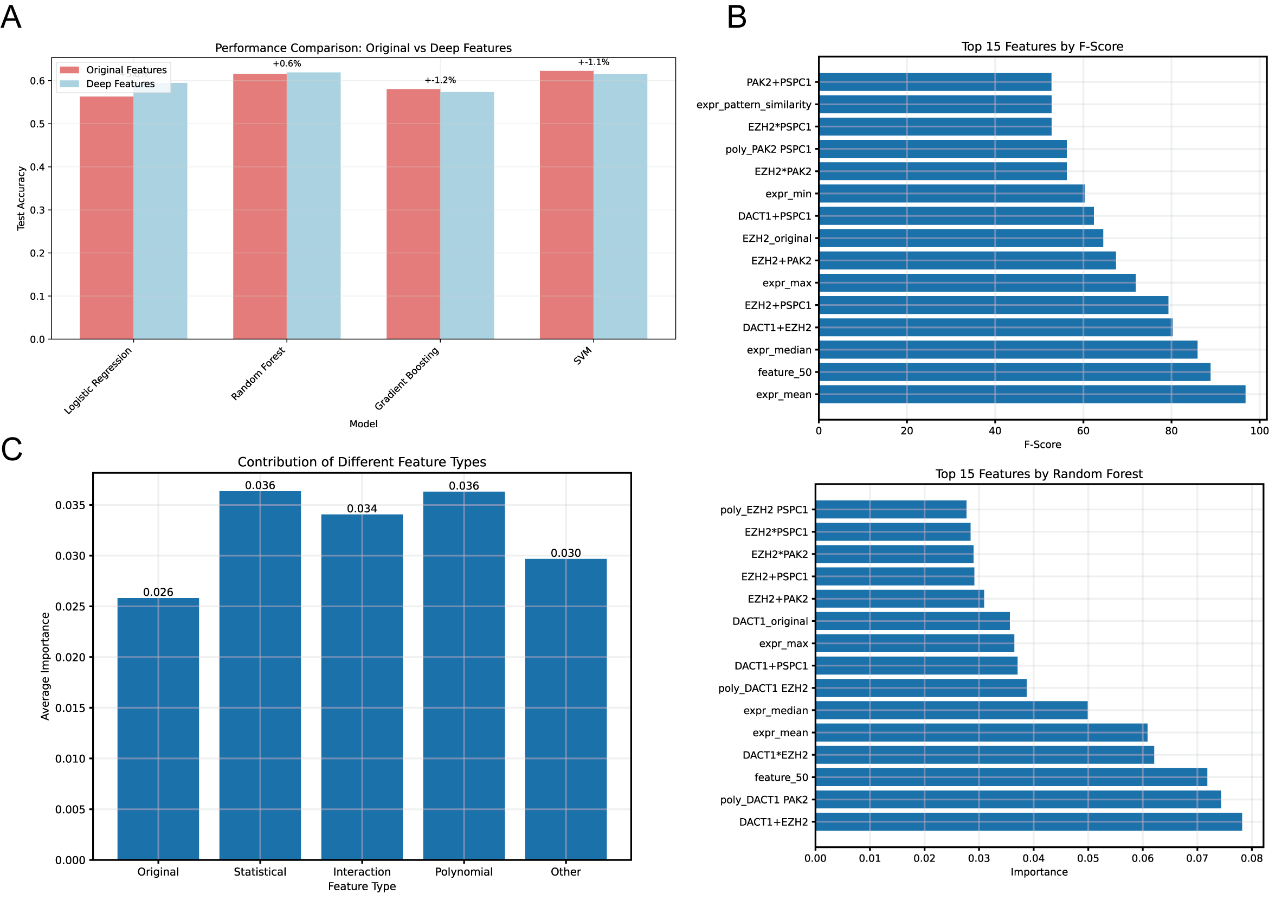


**Supplementary Figure 6 Effect of deep feature engineering on gastric cancer staging prediction** (A) Comparison of model performance between raw features and deep features. The accuracy of four models, logistic regression, random forest, gradient boosting and SVM, when using raw and deep features is demonstrated. (B) Ranking of the top 15 important features by F-Score score. (C) Comparison of the contribution of different feature types (raw, statistical, interactive features, polynomial, and other) to the model. (D) Ranking of the top 15 important features evaluated by Random Forest.


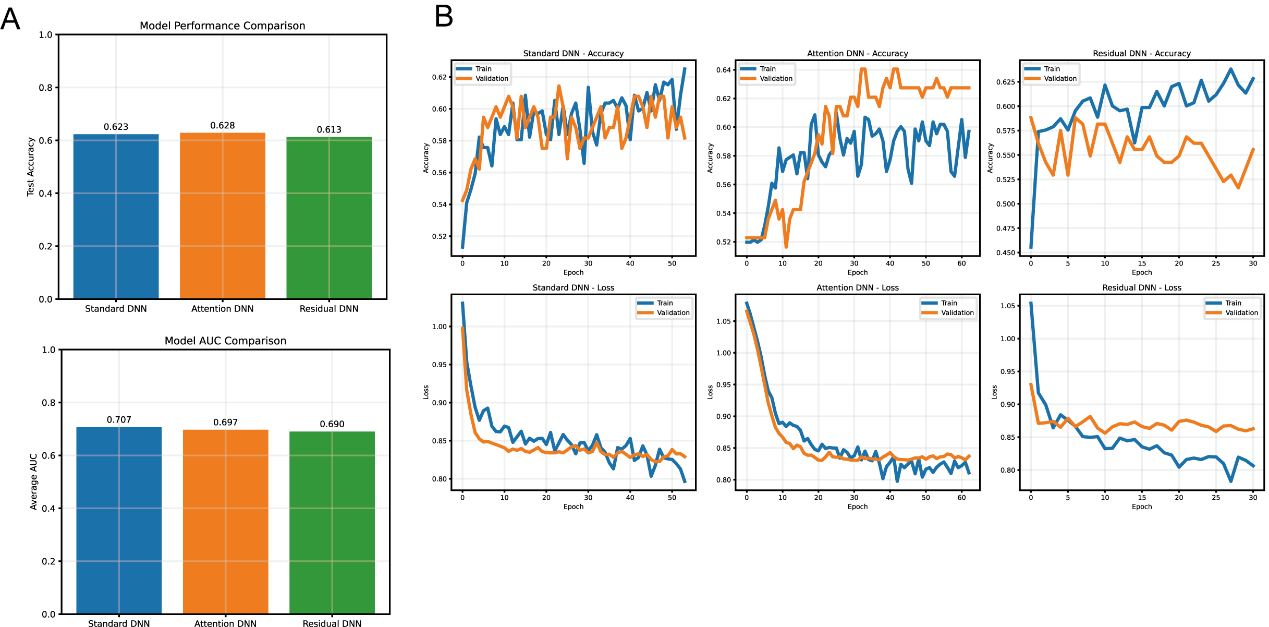


**Supplementary Figure 7 Integration analysis of deep feature engineering with deep learning models** (A) Performance comparison of three deep neural network architectures with AUC values. The top panel shows the model accuracy comparison and the bottom panel shows the corresponding AUC scores. (B) Accuracy and loss function change curves during model training. The top row shows the accuracy changes of the three models on the training and validation sets, and the bottom row shows the corresponding loss function changes. The training dynamics of the three architectures, standard DNN, attention DNN and residual DNN, are demonstrated.


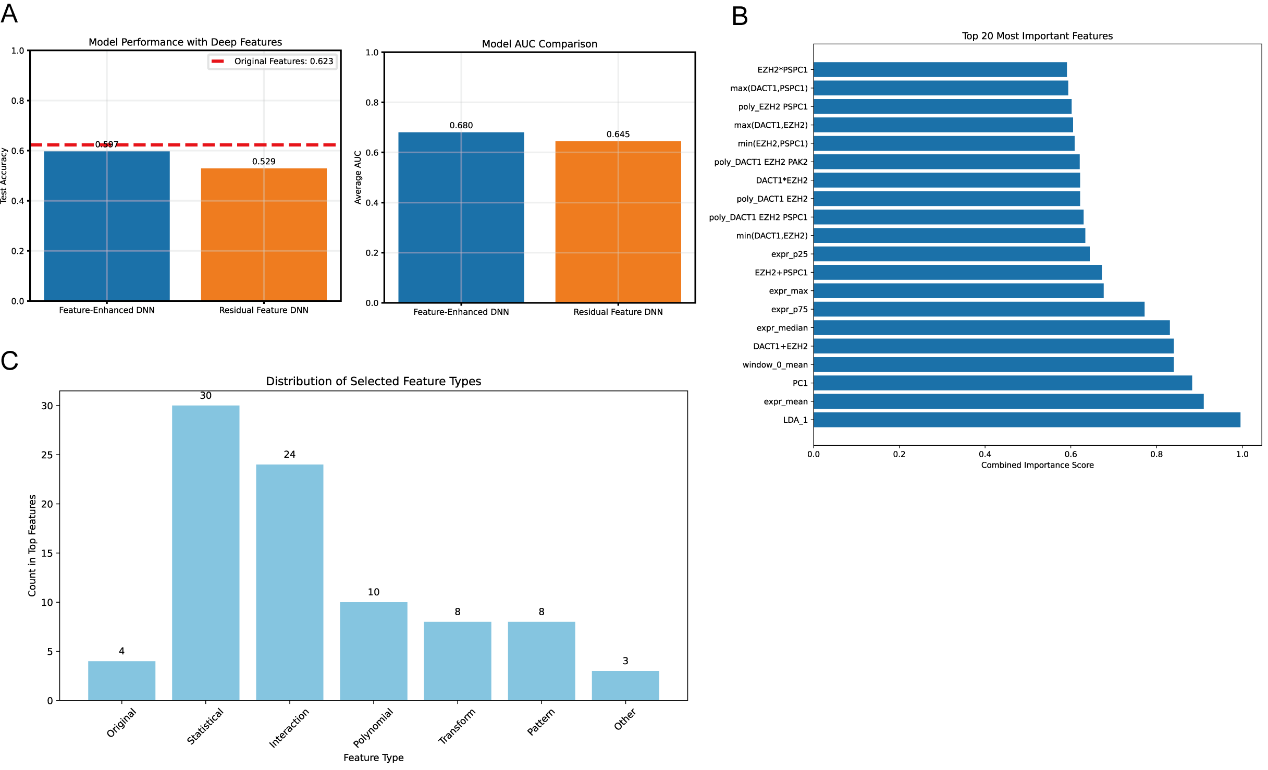


**Supplementary Fig. 8 Integration evaluation of deep feature engineering with deep neural networks** (A) Comparison of model performance and AUC between feature-enhanced DNN and residual feature DNN. The red dashed line indicates the baseline performance level. (B) Contribution ranking of the top 20 most important features generated by deep feature engineering. (C) Distribution of different feature types showing the number of raw features, statistical features, interaction features, polynomial features, transformed features, pattern features and other features in the selected feature set.


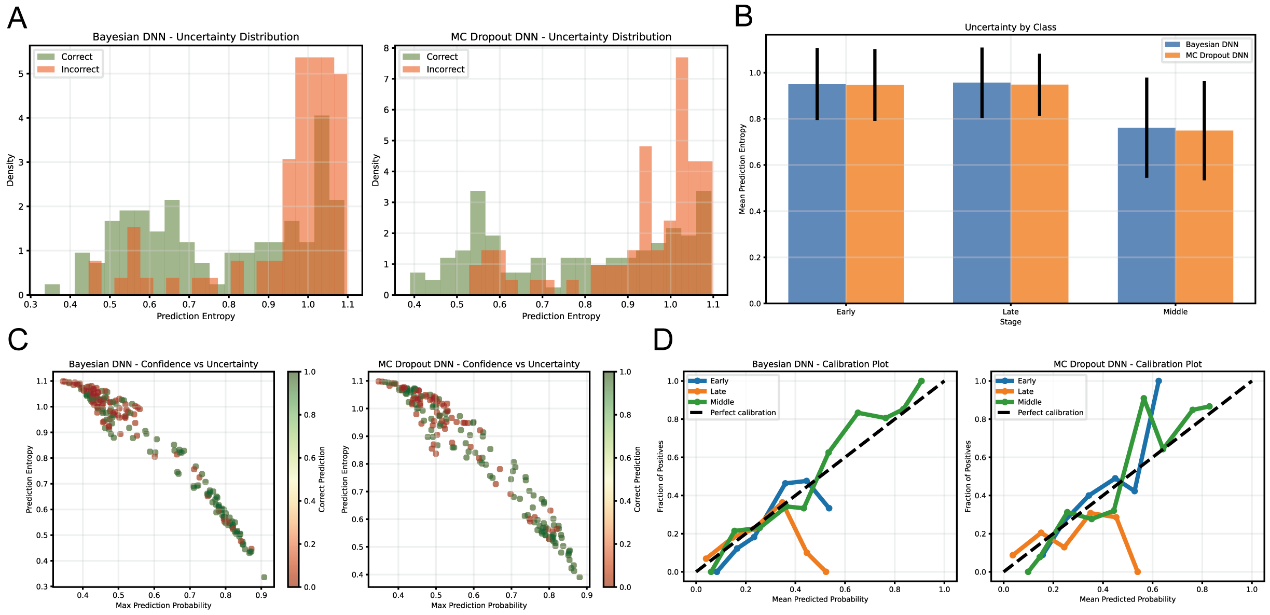


**Supplementary Fig. 9 Quantitative uncertainty analysis of Bayesian deep neural networks** (A) Uncertainty distribution of Bayesian DNN and MC Dropout DNN. Predictive entropy distributions for correct predictions (green) and incorrect predictions (orange) are shown. (B) Comparison of uncertainty between Bayesian DNN and MC Dropout DNN at different staging (early, late, mid). (C) Scatter plot of confidence versus uncertainty for Bayesian DNN and MC Dropout DNN. The color of the dots indicates the actual staging and demonstrates the negative correlation between the maximum predictive probability and the predictive entropy. (D) Calibration curves of the two Bayesian methods. The calibration of the prediction probabilities for early (blue), late (green), and intermediate (orange) stages are shown, and the dashed line indicates perfect calibration.
