# Supplementary figures and images for: Liquid-liquid phase separation in gastric cancer: identifying novel biomarkers and therapeutic targets through gene signature analysis
Source: Front Immunol. 2025 Sep 1;16:1620390. doi: 10.3389/fimmu.2025.1620390 (PMC12434088; doi:10.3389/fimmu.2025.1620390)

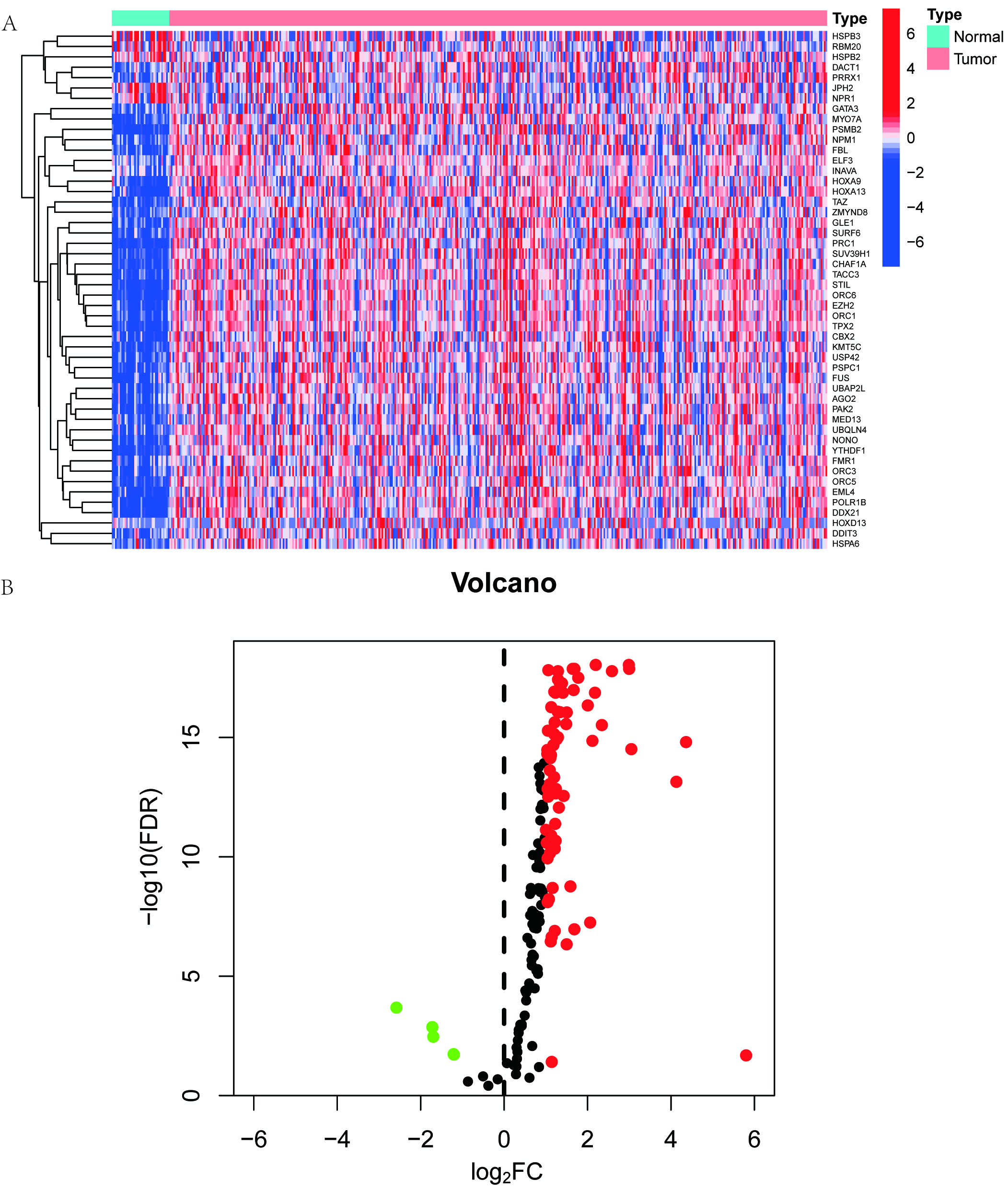

Supplement: Supplementary file 2 [file Image1.tif]

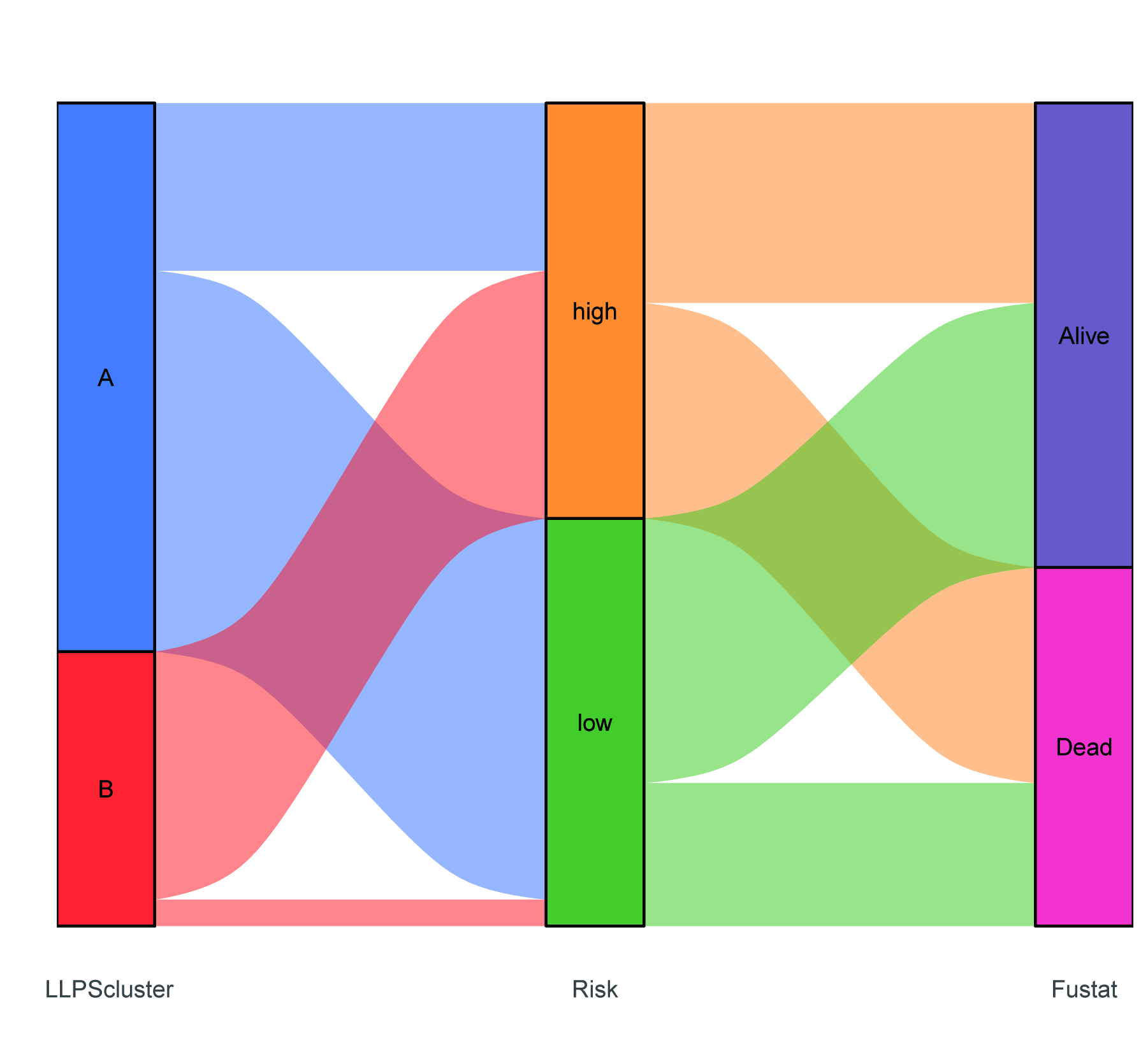

Supplement: Supplementary file 3 [file Image2.tif]

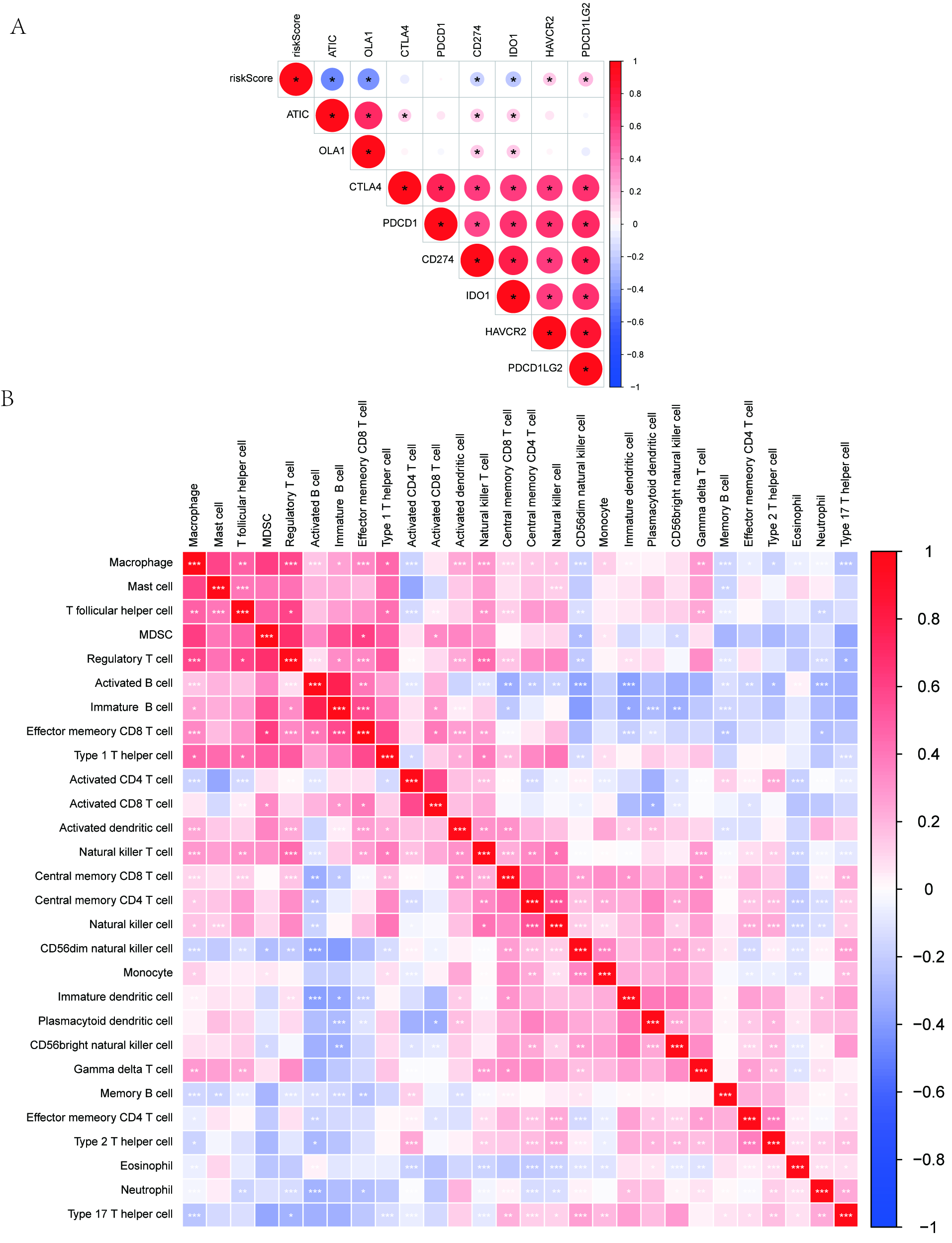

Supplement: Supplementary file 4 [file Image3.tif]
