# Supplementary material for: Liquid-liquid phase separation in gastric cancer: identifying novel biomarkers and therapeutic targets through gene signature analysis
Source: Front Immunol. 2025 Sep 1;16:1620390. doi: 10.3389/fimmu.2025.1620390 (PMC12434088; doi:10.3389/fimmu.2025.1620390)
Supplement: Supplementary file 5 [file Table1.doc]

**Table S1** PCR primer sequences.

| **Primer** | **（**5' to 3'） |
| --- | --- |
| PAK2-F | CACCCGCAGTAGTGACAGAG |
| PAK2-R | GGGTCAATTACAGACCGTGTG |
| DACT1-F | TGTGAATCCCAAGTACCAGTGT |
| DACT2-R | CCGTCAGACAAAGGAGAAACATT |
| EZH2-F | GTACACGGGGATAGAGAATGTGG |
| EZH2-R | GGTGGGCGGCTTTCTTTATCA |
| PSPC1-F | TGTGGATGATCGCGGTAGAG |
| PSPC1-R | CGAGGGGTCGTTGTTAGCA |
| GAPDH-F | TTGCCCTCAACGACCACTTT |
| GAPDH-R | TGGTCCAGGGGTCTTACTCC |
