# Supplementary material for: Liquid-liquid phase separation in gastric cancer: identifying novel biomarkers and therapeutic targets through gene signature analysis
Source: Front Immunol. 2025 Sep 1;16:1620390. doi: 10.3389/fimmu.2025.1620390 (PMC12434088; doi:10.3389/fimmu.2025.1620390)
Supplement: Supplementary file 6 [file Table2.doc]

Table S2： Antibody information.

| Antibodies | Company | Catalog Number | Host | Working Concentratio |
| --- | --- | --- | --- | --- |
| GAPDH | ProteinTech | 60004-1-Ig | Mouse | 1:200000(WB) |
| DACT1 | ProteinTech | 27237-1-AP | Rabbit | 1:2000(WB);1:200(IF) |
| PSPC1 | ProteinTech | 16714-1-AP | Rabbit | 1:5000（WB）;1:500(IF) |
| EZH2 | selleck | F0281 | Rabbit | 1:1000(WB) |
| PAK2 | selleck | F1046 | Rabbit | 1:5000（WB） |
